# A review of the biological and clinical implications of RAS-MAPK pathway alterations in neuroblastoma

**DOI:** 10.1186/s13046-021-01967-x

**Published:** 2021-06-08

**Authors:** Vid Mlakar, Edouard Morel, Simona Jurkovic Mlakar, Marc Ansari, Fabienne Gumy-Pause

**Affiliations:** 1 CANSEARCH Research Platform for Pediatric Oncology and Hematology, Department of Pediatrics, Gynecology and Obstetrics, Faculty of Medicine, University of Geneva, Avenue de la Roseraie 64, 1205 Geneva, Switzerland; 2 Division of Pediatric Oncology and Hematology, Department of Women, Child and Adolescent, University Hospital of Geneva, Rue Willy-Donzé 6, 1205 Geneva, Switzerland

**Keywords:** Neuroblastoma, RAS-MAPK, ALK, RAS, MEK1/2, ERK1/2, RASopathie, Inhibitors

## Abstract

Neuroblastoma is the most common extra-cranial solid tumor in children, representing approximately 8% of all malignant childhood tumors and 15% of pediatric cancer-related deaths. Recent sequencing and transcriptomics studies have demonstrated the RAS-MAPK pathway’s contribution to the development and progression of neuroblastoma. This review compiles up-to-date evidence of this pathway’s involvement in neuroblastoma. We discuss the RAS-MAPK pathway’s general functioning, the clinical implications of its deregulation in neuroblastoma, and current promising therapeutics targeting proteins involved in signaling.

**Supplementary Information:**

The online version contains supplementary material available at 10.1186/s13046-021-01967-x.

## Background

Neuroblastoma (NB) is the most common extra-cranial solid tumor in children, representing approximately 8% of all malignant childhood tumors and 15% of pediatric cancer-related deaths [1, 2]. Most NB cases are sporadic (98%) and occur in very young children, with 90% of patients being younger than 5 years old at diagnosis and the median age being 19 months old [1–3]. This cancer originates in the sympathetic nervous system, most frequently in the adrenal medulla or the sympathetic nerve chain. Although the primary tumor is typically located in the abdomen, neck, chest, or pelvis, the most frequently observed metastatic locations are regional lymph nodes, bone marrow, bones, and subcutaneous tissues [1–6].

NB is a complex, highly heterogeneous disease with very significant variability in prognosis, ranging from spontaneous regression to highly aggressive and resistant disease despite multimodal therapy. Biologically, NB is not characterized by a high rate of mutations but rather by frequent recurrent chromosomal aberrations, some of which can be used as genetic markers providing prognostic information: whole-genome duplication is usually associated with a good outcome, whereas segmental chromosomal aberrations like *MYCN* amplification (MNA) and 11q deletion are commonly associated with a poor prognosis [6, 7]. Based on multiple prognosis factors, such as age at diagnosis, the stage of the disease, histology, grade of differentiation, *MYCN* status, genomic profile, and ploidy, the International Neuroblastoma Risk Group (INRG) classification system [6, 8] determines four main categories of patients: very-low-risk, low-risk, intermediate-risk, and high-risk NB patients have estimated 5-year event-free survival rates of > 85%, > 75% to < 85%, > 50% to < 75%, and < 50%, respectively [8]. High-risk NBs are the most frequently observed, representing approximately 40% of NBs [8]. Although the majority of high-risk NB patients respond positively to an initial intensive multimodal therapy including surgery, high-dose chemotherapy with autologous bone marrow transplantation, radiotherapy, and immunotherapy, half of them will relapse. Relapse usually occurs within 2 years of the end of the initial treatment and is rarely curable. This very poor outcome clearly requires new therapeutics (see the recent review on NB by Matthay et al. [6]).

The *ALK* gene came to prominence in 2008 after its mutation was detected in the majority of cases of familial NB [9, 10]. It was found to be the most common somatically mutated gene in primary NB [11–13], particularly in the high-risk category [9, 14, 15]. Very recently, kinases in the RAS mitogen-activated protein kinase (RAS-MAPK) pathway, one of the downstream signal transduction pathways activated by ALK, were also reported to be frequently mutated in NB. This was especially the case in relapsed samples, in which activating mutations of this pathway were detected in almost 80% of these highly therapy-resistant tumors [16]. These results provide a rationale for using RAS-MAPK pathway alterations as biomarkers for novel targeted treatment approaches for NB.

This review describes the latest knowledge about RAS-MAPK pathway alterations in NB and provides a better understanding of its contribution to tumor development and progression; it also addresses current knowledge about using RAS-MAPK pathway inhibitors in NB treatment.

## Methodology

### Selection of RAS-MAPK genes and the analysis of genes associated with NB and the RAS-MAPK pathway

**Fig. 1:**
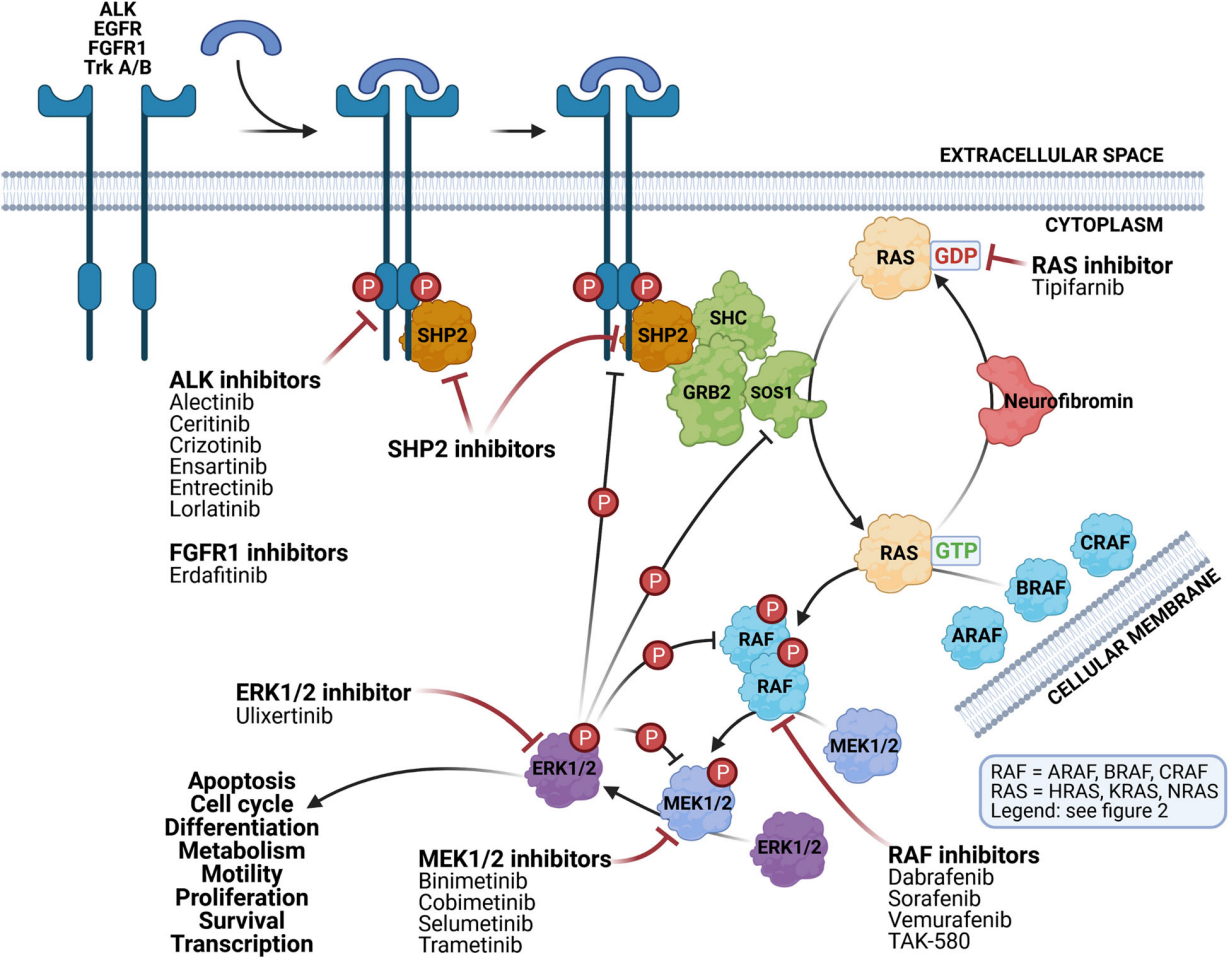
Schematic representation of the RAS-MAPK signaling pathway

**Fig. 2:**
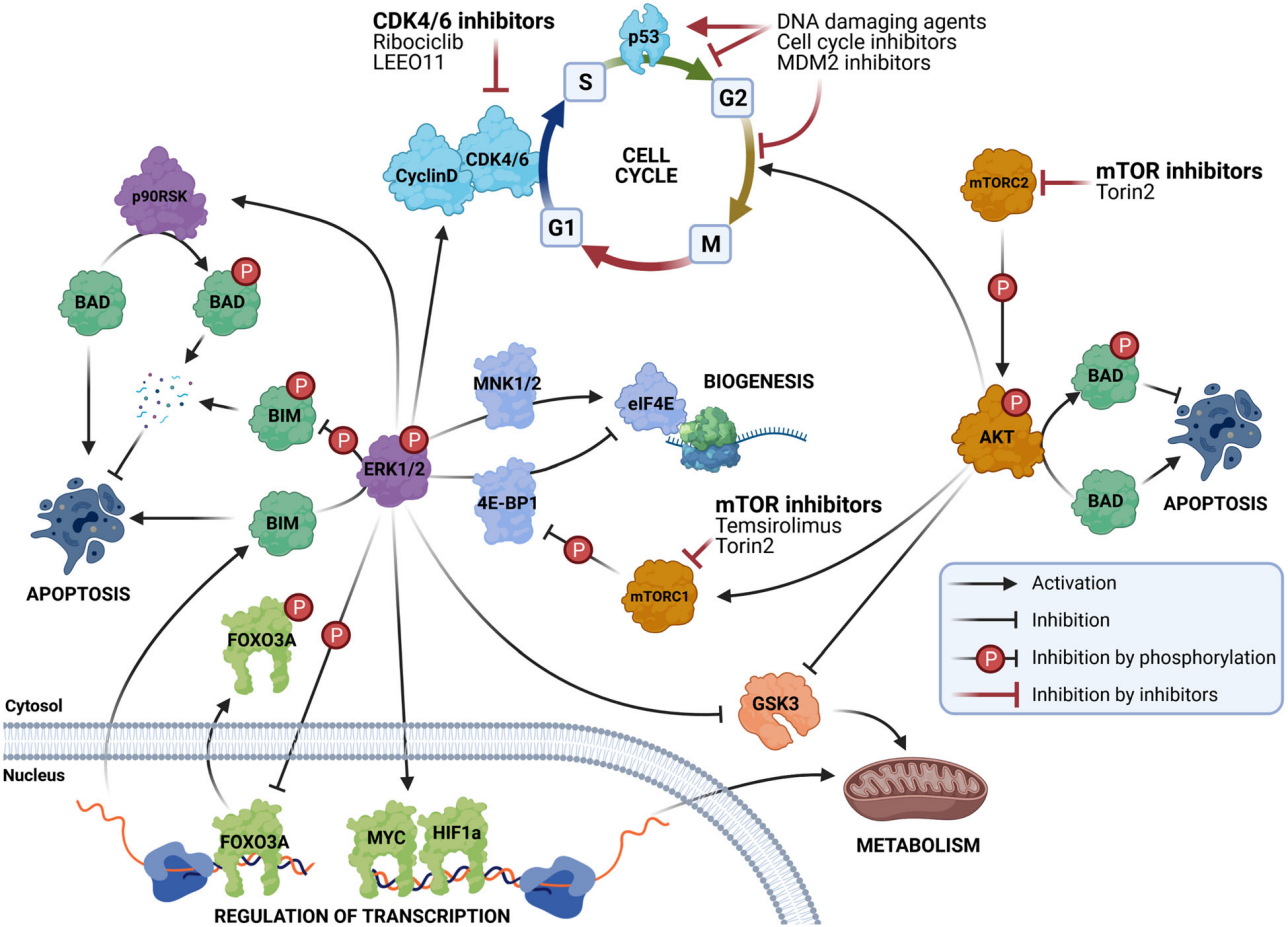
Schematic and simplified representation of the biological consequences of the RAS-MAPK signaling pathway

QIAGEN’s Ingenuity Pathway Analysis software (IPA, QIAGEN, Redwood City, USA) was used to plot the established RAS-MAPK pathway and retrieve the references describing its interactions. The genes associated with NB were extracted from publications by Tolbert et al. [17] and Tonini et al. [18]. Next, IPA software (QIAGEN, Redwood City, USA) was used again to screen for references describing the associations between those genes and the RAS-MAPK pathway (Table 1). Figures 1 and 2 were created using BioRender.com.

**Table 1:** Frequency of mutations in the RAS-MAPK genes of different adult and childhood cancers (source: COSMIC database, April 6th, 2020)

Level	Gene	Mutations	Neuroblastoma (%)	Brain (%)	Breast (%)	Colorectal (%)	Blood (%)	Lung (%)	Skin (%)
**Receptor**	***ALK***	F1174L, R1275Q, R1245V	6.3	0.8	7.3	5.7	5.6	4.4	9.1
**Protein adaptor**	***SHC1***	E343D	0.1	0.1	0.6	2.0	0.2	0.7	1.0
	***GRB2***			0.4	2.2	0.6	1.0	0.3	0.9
	***PTPN11***	Mutations in SH2 and PTP domains	1.0	1.7	1.3	1.7	4.7	0.9	2.4
**SOS**	***SOS1***	N993Sfs*5	0.1	0.7	3.3	3.5	1.3	2.1	3.7
**RAS**	***N-RAS***	Q61K/E/L, G13R, A59T	0.7	1.0	0.5	3.7	9.5	0.8	15.0
	***K-RAS***	G12V		0.8	1.4	32.4	4.9	14.8	2.8
	***H-RAS***	Q61K	0.1	0.0	0.6	0.9	0.2	0.5	10.6
**RAF**	***BRAF***	V600E. F595L. R719P	0.1	4.7	2.1	12.3	8.2	2.2	41.0
	***RAF1***	L397V	0.1	0.3	1.6	2.3	0.6	1.0	3.1
	***ARAF***			0.2	0.6	2.0	0.3	1.4	1.9
**MEK1/2**	***MAP2K1***	K57N		0.3	1.8	1.9	2.2	0.7	4.7
	***MAP2K2***	c.920-66G > T	0.1	0.1	0.8	1.3	0.5	0.6	2.1
**ERK1/2**	***MAPK3***	E367D	0.1	0.1	0.4	1.0	0.2	0.3	1.4
	***MAPK1***			0.4	1.6	1.3	1.1	0.3	1.5
**NF**	***NF1***	Inactivating nonsense mutations	1.2	7.2	6.1	9.5	3.5	7.2	16.8

### Analysis of publicly available data

The COSMIC database was used to collate the mutation data on seven different tumor types: blood, brain, breast, colorectal, lung, neuroblastoma, and skin cancers. Data were extracted for thoracic NB and all NB, and then they were pooled by adding the number of analyses and identified mutations.

### Analysis of RAS-MAPK inhibitors in clinical trials

The clinicaltrials.gov database was used to screen for all active, recruiting, or completed phase I to III clinical trials using molecules targeting the RAS-MAPK pathway in NB patients (Table 1, Figs. 1 and 2).

## The clinical implications of ALK and RAS-MAPK pathway alterations in NB

### The implications of ALK and the RAS-MAPK pathway in primary NB tumors

Many different studies have reported *ALK* mutations in approximately 6–10% of sporadic primary NB tumors and around 12–14% of the high-risk category [9–11, 15]. To understand the relative importance of *ALK* in the development of NB we compared the frequency of *ALK* mutations in NB with the frequencies of *ALK* mutations in other tumor types. *ALK* is found to be mutated more frequently in NB than in brain, colorectal, lung, and blood cancers. Only breast and skin cancers have higher *ALK* mutation rates than NB, suggesting that *ALK* mutations play a more significant role in NB development than in other common cancer types (Table 1).

*ALK* mutations in NB are mainly single amino acid substitutions; in 90% of cases they are located in the kinase domain, with hot spots at amino acids F1174, R1275, and F1245 [9–11, 15]. Interestingly, in approximately 50% of NB cases, *ALK* mutation is found together with *MYCN* amplification and correlated to poor prognosis [11].

Interestingly, in an additional 2–3% of NB samples, ALK was found to be activated by gene amplification, increasing both protein expression and activity [19–21]. *ALK* amplification, which is mutually exclusive of point mutation, is also almost always associated with *MYCN* amplification, with both genes located in the same locus (2p23–24, Table 2) and associated with poor prognosis [11, 13, 15, 22].

**Table 2:**
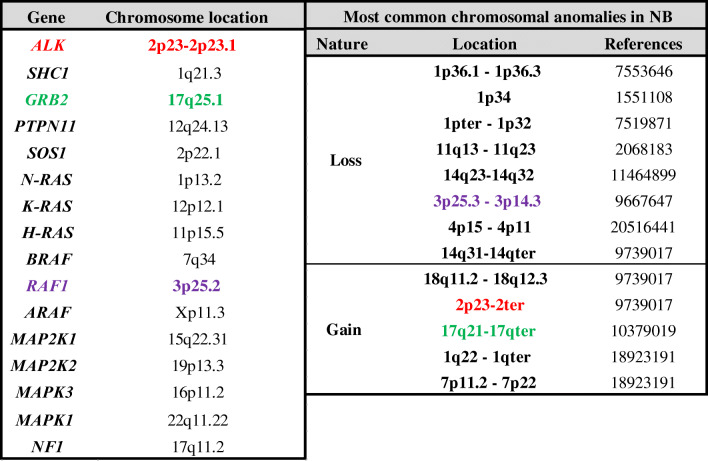
Chromosomal locations of the RAS-MAPK pathway genes and most common chromosomal anomalies in NB

References: PMID

Genes in color are located at chromosomal anomalies

Although *ALK* mutations have been observed in all clinical risk groups, a recent study analyzing a large cohort of more than 1500 NB patients demonstrated that the presence of an *ALK* mutation was independently correlated with survival: patients with an *ALK* mutation showed a 1.4-fold greater risk of an event within 5 years than patients without one. Statistically significant correlations were independently observed for all the groups of *ALK* mutations: *ALK* aberration, *ALK* copy number gain, and *ALK* amplification [15]. For a more general review of *ALK* and NB, we recommend the recent review by Trigg et al. [23].

Different studies have also reported somatic RAS-MAPK pathway gene alterations (mutation, amplification, or deletion) leading to the activation of this pathway in large cohorts of sporadic NB patients, with a frequency of around 4–10% [21, 24, 25]; the most frequently mutated genes were *PTPN11* (2.9%), *NRAS* (0.8%), and *NF1* [14, 24]. Typically, the first mutations observed in *NRA*S were the known activators Q61K and C181A, which were initially discovered in SK-N-SH and SK-N-AS cell lines, respectively [16, 26–28]. Also, *HRAS* and *KRAS* can be mutated, notably by G12D and Q61K substitution, for instance [16, 29].

Most recently, Ackerman et al. sequenced the genomes of 418 pre-treated NB. In line with a previous study, they found that 9% had *ALK* mutations, but also that 6% had alterations in the RAS-MAPK pathway genes (*NF1*: 1.2%; *HRAS*: 1%; *PTPN11*: 1%: *NRAS*: 0.5%; *BRAF*: 0.5% *KRAS*: 0.2%; and *FGFR1*: 0.2%). ALK-RAS pathway alterations were detected in all NB risk categories and were found to be strongly correlated to poor outcomes, even in the high-risk category [25]. Interestingly, patients harboring RAS pathway mutations were found to have a worse prognosis than those with *ALK* mutations. Furthermore, Ackermann et al. also investigated the relationship between ALK-RAS alterations and telomere maintenance mechanisms (defined by *MYCN*, *TERT*, and APB alterations). Although the presence of that telomere maintenance mechanism was associated with worse outcomes compared to those without those mechanisms, an additional mutation in the ALK-RAS pathway increased NB aggressiveness, resulting in a higher mortality rate. In contrast, in NB patients without a telomere maintenance mechanism, the presence of an ALK-RAS alteration did not affect patient outcomes [25]. Eleveld et al. were recently able to define an mRNA signature, consisting of six genes (*ETV4*, *ETV5*, *DUSP6*, *MAFF*, *ETV1*, and *DUSP4*) that was correlated with an increase in RAS-MAPK pathway activity. A high expression of these signature genes was associated with poor survival in primary NB but also with MEK1/2 and ERK1/2 phosphorylation and sensitivity to different MEK1/2 inhibitors in cell lines [30].

Mutations in the genes coding for the RAF family of proteins are very rare in NB. *BRAF* aberrations have been observed in 1% of NB cases, whereas the study by Shukla et al. reported no alterations in *ARAF* and *CRAF* [26]. However, there was a greater expression of all the RAF proteins in the high-risk NB category [31]. The main mutation found in *BRAF* is the V600E substitution [26]. This mutation is located in BRAF’s kinase domain and leads to a constitutively active form of BRAF [32]. Interestingly, a tandem duplication in the *BRAF* gene was also detected in a relapse NB. This duplication resulted in the expression of a *BRAF* transcript that encodes a protein with two kinase domains. Accordingly, the protein was twice as active, which made the RAS-MAPK signaling pathway more active [16].

A *MEK1/2* mutation is a very rare event in NB, with a frequency of 0.5% [26]. In one NB sample, a *MEK1* somatic activating mutation (K57N) was observed, located between the protein’s nuclear export signal and its kinase domain. This genetic lesion leads to constitutive activation of the RAS-MAPK pathway in vitro [33]. On the contrary, no reports of *ERK* mutations have been found in NB.

Comparisons of NB with six other major cancer types showed significant differences in the frequency of mutations in the major RAS-MAPK pathway genes. NB, brain and breast tumors do not demonstrate high mutation frequencies along this pathway (Table 1), while colorectal, blood, lung, and skin cancers have high frequencies in at least one of the RAS-MAPK pathway genes. Interestingly, two genes, *RAF1* (3p25.2) which encodes for the CRAF protein, and GRB2 (2p23-2p23.1), were found to be located in regions that sustain frequent chromosomal abnormalities in NB (Table 2).

Finally, different studies have also reported alterations in genes associated with the RAS-MAPK pathway, such as *CDK4*, *LIN28B*, *CCND1*, *SMO*, *SOS1*, *CIC*, *DMD*, and *DUSP5* [24, 25, 30, 34, 35].

### ALK and RAS-MAPK mutations at relapse

Different studies have reported that *ALK* mutations were more frequently detected in NB relapse samples, with a frequency of 15–40% [16, 24, 36]. In 2015, Eleveld et al. analyzed 23 paired diagnostic and relapsed NB samples. They were able to show not only a clonal evolution from diagnosis to relapse sample but also that 78% (18/23) of the relapsed NB samples harbored a genetic aberration predicted to activate the RAS-MAPK pathway (*ALK* (10/23); *NF1* (2/23); *PTPN11* (1/23); *FGFR1* (1/23); *NRAS* (1/23); *KRAS* (1/23); *HRAS* (1/23); and *BRAF* (1/23)) [16]. In line with these results, Padovan-Merhar et al. reported that suspected *ALK* driver mutations were present in 7% (3/43) of samples at diagnosis, 17% (7/41) of the post-treatment samples, and 20% (11/54) of the samples at relapse. Furthermore, they observed more suspected oncogenic *ALK* mutations in relapsed disease than at diagnosis, as well as enrichment of RAS-MAPK pathway mutations at relapse [24]. Also, single-nucleotide variants (SNVs) in RAS proteins are more frequent in relapsed NB, notably, for instance, in *HRAS* with the somatic Q61K mutation [16, 24, 26], resulting in the constitutively active form of RAS due to inactivation of the GTPase domain [37].

### Familial NB, sporadic NB germline susceptibility genes, and *RASopathies*

Familial cases of NB are very rare, representing 1–2% of all NB cases, and they are inherited in an autosomal-dominant manner. In recent years, many advances have been made in our genetic comprehension of these cases. *PHOX2B* (4p12), a gene known *to be* an essential regulator of the development of a normal autonomic nervous system and frequently associated with neurocristopathies, was the first to be identified in this setting in 2004 [38, 39]. However, it was only detected in a small subset of familial NB (approximately 10%). In 2008, two different, independent studies reported *ALK* (2p23) as a gene involved in more than half of familial NB cases. Additional studies finally reported *ALK* germline mutations in almost 80% of these families [15, 17, 40]. The majority of these mutations are located in the *ALK* tyrosine kinase domain, resulting in constitutive activation of the kinase [9, 10]. The penetrance is variable, highly related to the activating effect of the mutation [15], but estimated at around 50% overall [41]. As yet, no other genes have been identified to explain the 10% of familial cases without the *PHOX2B* or *ALK* germline mutations.

Interestingly, NB susceptibility genes were recently identified in the germline DNA of a patient with sporadic NB, suggesting that common germline polymorphisms with low penetrance (i.e., *NBAT-1*, *CASC15*, *BARD1*, *LMO1*, *HSD17B12*, *DUSP12*, *LIN28B*, *HACE1*, *SPAG16*, *NEFL*, *MLF1/RSRC1*, *CPZ*, *CDKN1B*, *SLC16A1*, *MSX1, MMP20, KIF15*) or more rare variants with higher penetrance (i.e., *ALK*, *BARD1*, *CHEK2*, *AXIN2*, *TP53, APC, BRCA2, SDHB, SMARCA4, LZTR1, BRCA1*) may also have a relevant role in NB carcinogenesis [17, 18]. Remarkably, when investigating the associations between these genes and the RAS-MAPK pathway, many of them were reported to interact with this pathway either in NB or other cancers (Table 3). This further supports the view that the RAS-MAPK pathway is one of the central targets in the process of tumorigenesis.

**Table 3:** Interactions of the RAS-MAPK pathway with NB associated genes

	Candidate genes	Mutation	Location	RAS-MAPK involvement	References
**Low penetrance genes**	CASC14/NBAT-1	Loss of function	6p22	MAPK1/3 inhibition	[1, 2]
	CASC15		6p22		
	BARD1	Gain of function	2q35	MAPK1^a^	[3–6]
	LMO1	Gain of function	11p15.4	MAPK1/3 activation	[7, 8]
	HSD17B12		11p11.2		
	DUSP12	NA	1q23.3	MAPK1, HRAS inhibition	[9–12]
	LIN28B	Gain of function	6q16	MAPK1/3 activation	[13, 14]
	HACE1	Loss of function	6q16	MAPK1/3 inhibition	[13, 15, 16]
	SPAG16		2q34		
	NEFL		8p21		
	MLF1/RSRC1	NA	3q25	ARAF^a^	[17]
	CPZ		4p16		
	CDKN1B	Inhibited by GRB2 through p27Kip1	12p13.1	MAPK1	[16, 18–22]
				GRB2	
	SLC16A1	NA	1p13.2	KRAS^a^	[23–27]
				NRAS^a^	
				HRAS^a^	
	MSX1	Gain of function	4p16.2	ARAF activation	[28, 29]
	MMP20	NA	11q22.2	Activation of pathway	[30]
	KIF15	NA	3p21.31	Activation of pathway	[31–33]
**High penetrance genes**	CHEK2	Loss of function	22q12.1	MAPK1/3 inhibition	[5, 34, 35]
	AXIN2		17q24.1		
	BRCA2	Loss of function	13q13.1	GRB2	[36–39]
	SDHB		1p36.13		
	SMARCA4	Loss of function	19p13.2	NF1^a^	[40–42]
				MAPK1^a^	
	LZTR1	Loss of function	22q11.21	NF1	[23, 25, 26, 43, 44]
				NRAS inhibition	
	BRCA1	Loss of function	17q21.31	NF1	[45–48]
				MAPK3	
				MAPK1	
	NBPF23		1q21.1		
	SEZ6L2/PRRT2		16p11.2		
	APC	Loss of function	5q22.2	MAP2K1 inhibition	[39, 49]
	TP53	Loss of function	17p13.1	MAPK1	[50–83]
				HRAS	
				GRB2	
				KRAS	
				BRAF	
				SHC1	
				PTPN11	
				MAP2K1	
				MAP2K2]	
				ARAF	

For references 1 to 83 in this table, please see Supplementary Material list 1

*NA* Information not available

^a^Putative interaction with RAS-MAPK pathway modeled by IPA

Additionally, NB has been reported to be associated with diseases well-known to predispose cancer, such as Li Fraumeni syndrome (*TP53* mutations) [42], familial paraganglioma/pheochromocytoma (*SDHB* mutations) [43], and Beckwith–Wiedemann syndrome (*CDKN1C* mutations or loss of expression) [44]. It has also been associated with other rare syndromic diseases, more particularly with those characterized by the presence of germline mutations located along the RAS-MAPK pathway. This group of diseases, also known as *RASopathies*, includes neurofibromatosis 1 (*NF1* mutations), Costello syndrome (*HRAS* mutations), Noonan syndrome, and such Noonan-like syndromes as Noonan syndrome-like disorder with loose anagen hair and the LEOPARD syndrome (mutations in *PTPN11*, *SOS1*, *KRAS*, *NRAS*, *RAF1*, *BRAF*, *MEK1*, *SHOC2*, *MEK2*, *RIT1*, *and CBL*) [41, 45–47].

## Biology of the RAS-MAPK pathway

The RAS-MAPK pathway’s importance is not only underpinned by its clinical relevance as displayed through its frequent dysregulation in cancerous cells. The pathway is firmly embedded in normal cell function as one of the central signaling axes that affect many different cellular functions (Figs. 1 and 2).

For example, the RAS-MAPK’s ERK1/2 end kinase is known to regulate the transcription of several hundred genes through its activation of a vast array of transcription factors. Through ERK1/2 regulation of transcription, RAS-MAPK regulates the progression of cells from the G1 to the S phase by activating expression of Cyclin D [48, 49]. Interestingly, Cyclin D1 is also a well-established NB oncogene that is associated with 11q13.3 gains [50]. Another prominent example of the importance of transcription regulation is the inhibition of apoptosis through ERK1/2-mediated repression of BIM, which is one of the most potent members of the BH3-only proteins responsible for the inhibition of antiapoptotic proteins such as BCL-2, BCL-xL, MCL1, BCL-W, and A1 [51]. Inhibition of BIM happens not only through direct phosphorylation but also through the inhibition of transcription by ERK1/2 phosphorylation of the FOXO3A transcription factor [52]. Phosphorylation of FOXO3A promotes its expulsion form nucleus and subsequent proteasomal degradation [52]. Metabolic switching, increased production of lactate, and a decrease in oxidative phosphorylation are other consequences of an activated RAS-MAPK pathway [53, 54]. The key targets responsible for metabolic adaptation are the phosphorylation of MYC and HIF1α [55–57], which are known to regulate the transcription of genes involved in glucose metabolism and respiration [53, 58]. Recently, it has been demonstrated that ERK1/2 is involved in cell differentiation through the transcriptional repression of key pluripotency transcription factors, such as NANOG, KLF2, and KLF4 [59], and through crosstalk with LIF and BMP signaling through STAT3 [60, 61].

The regulation of gene expression, however, is exerted not only at the level of transcription but also at the level of translation. ERK1/2, for example, is known to interact with MNK1/2 [62], which in turn activates eIF4E, a known activator of translation [63]. Interestingly, this process is regulated by a feedback loop, through MSK1/2, which activates an eIF4E inhibitor, 4E-BP1 [64].

Although the regulation of transcription is the major target of RAS-MAPK pathway signaling, RAS-MAPK also regulates cellular functions through the direct phosphorylation of its targets. ERK1/2 is also known to inhibit apoptosis by direct phosphorylation of the pro-apoptotic factor BIM, mentioned above [65]. Through ERK1/2, RAS-MAPK is also involved in the response to oxidative stress and, together with phosphorylated Hsp27, it is necessary for the proteasome degradation of BIM and the inhibition of apoptosis [66]. p90RSK, which controls apoptosis activation through interaction with BAD, is another important modulator of apoptosis that is a target of ERK1/2 [67]. Finally, cellular mobility is also directly affected by ERK1/2, which is known to regulate the rate and polarity of actin polymerization [68, 69] and to promote the turnover of focal adhesion [70–72]. For more information on RAS-MAPK signaling’s effects on cellular biology, we suggest a recent review by Lavoie et al. [64].

### ALK and other RTKs involved in RAS-MAPK activation

The RAS-MAPK pathway is usually activated by receptor tyrosine kinase (RTK) (Fig. 1). As discussed above, ALK is the most studied RTK in NB. However, other RTKs are known to activate the RAS-MAPK pathway and have sometimes been found to be mutated in relapsed NB. These include FGFR1 (fibroblast growth factor receptor 1), EGFR (epidermal growth factor receptor) [24], and Trk A and B [73]. The main aberration found in FGFR1 [50] is the somatic N546K mutation [16], which is located in the kinase domain and alters auto-phosphorylation, leading to an increase in its kinase activity [74]. Because the present review focuses on the RAS-MAPK pathway, we refer the reader to other excellent general reviews of RTKs [75] and their involvement in NB [23, 76].

### RAS activation and RAS associated proteins

Activated RTKs’ first targets are the three RAS proteins—KRAS, NRAS, and HRAS (Fig. 1). The activation is a complex sequence of events, involving the ultimately inactive RAS being converted into its active form by the addition of GTP. At least three adaptor proteins—SHP2 (PTPase encoded by *PTPN11*), Shc, and Grb2—are required for the first step in the signalization reaction [77]. This complex forms after RTK’s phosphorylation by the recruitment of PTPases which contain the SH2 motifs responsible for specific binding between PTPase and RTK. Next, PTPase becomes tyrosine-phosphorylated, which induces a significant change in its conformation and increases phosphatase activity [78, 79]. The change in conformation appears to be essential for the recruitment of the other two factors, Shc and Grb2 [80]. Nevertheless, SHP2 also appears to serve other functions, as some studies suggest that it dephosphorylates STAT3, an important transcription factor activated by terminal ERK1/2 kinase. After being recruited, Shc is activated by phosphorylation at Tyr317 [81]. Adaptor proteins are assembled on RTKs’ intracellular sites, and many known mutations lead to the constitutive association of both Shc and Grb2 proteins with RTK [77]. As already mentioned, mutations in the *PTPN11* gene are found in 3% of high-risk NBs [82]. The major mutation is the heterozygous A72T substitution located in the first SH2 domain of SHP2, converting SHP2 into a constitutively active phosphatase. After the assembly of Shc and Grb2 adaptor proteins, Sos1 protein is recruited to catalyze the addition of GTP to RAS [83]. Sos1 is a mammalian nucleotide exchange factor that, once recruited to the Shc/Grb2 complex by translocation to the plasma membrane, activates RAS through the exchange of GDP for GTP [84].

One of the most important aspects of RAS phosphorylation is its location. It has been demonstrated that the entire complex’s location with respect to cellular membranes facilitates the rate of exchange. The diffusion of proteins was shown to be insufficient for the reaction to happen [85]. Active transport is necessary for the subsequent transmission of the signal towards its targets. Although the sequences of all three RAS proteins are almost identical, they are located at different cellular compartments. KRAS is more abundant in cytoplasmic membranes, whereas HRAS is more common in intracellular vesicular membranes. These differences are presumably due to differential processing in the Golgi apparatus. Nevertheless, it has been demonstrated that Grb2a associates with both KRAS and HRAS, irrespective of their location [86].

In a physiologically normal state, cells have several means of controlling the activation and deactivation of RAS proteins. The first negative regulation to be described was the activation of the hydrolysis of RAS-bound GTP to GDP by p120GAP, neurofibromin, and GAP1. Neurofibromin, coded by the *NF1* gene, exerts its inhibitory action by stimulating the hydrolysis of RAS-GTP [87]. *NF1* seems particularly important in the development of NB as *NF1* aberrations have been found in primary NB and at an even higher frequency in relapsed NB samples [24, 88]. The *NF1* mutations observed in primary NB are typically SNVs, promoting the loss of neurofibromin’s activity. In relapsed tumors, however, we mostly observe *NF1* deletion. Depending on the NB cell lines, we observe either homozygous deletion of various sizes or heterozygous deletion with a splice site mutation in one of the alleles [16, 88]. In all of these situations, loss of neurofibromin results in the constitutively active form of RAS and thus over-signalization of the RAS-MAPK pathway.

The second negative regulation to be described was the feedback loop through the phosphorylation of Sos1 and the subsequent inactivation of RTK/Shc/Grb2/Sos1 by ERK1. The phosphorylation of Sos1 does not activate the dissociation of Sos1 from Grb2 but rather the dissociation of the entire Grb2/Sos1 complex from phosphorylated RTK [89]. It is currently unknown how much the deregulation of this feedback contributes to the activation of the RAS-MAPK pathway and to the development of NB or other cancer types.

### RAF activation

In normal cells, RAF proteins (ARAF, BRAF, and CRAF) represent the next step in the signaling cascade (Fig. 1). Although *BRAF* is one of the most frequently mutated genes in many cancers, it is interesting that BRAF does not directly interact with activated RAS. Signal transduction to the BRAF protein is accomplished by the heterodimerization between CRAF and BRAF that is dependent on the presence of RAS. That heterodimerization is also dependent on the phosphorylation of BRAF at the serine 621 and of the CRAF’s C terminus, whereas CRAF’s N terminus is not important for this interaction. On the other hand, CRAF’s association with RAS is independent of RAS activity as assessed by the dominant-negative form of RAS (G12V) or by the truncated RAS mutant lacking the COOH-terminal serine residues necessary for membrane localization [90, 91]. Interestingly, MEK1/2 phosphorylation can be achieved by CRAF alone, but it is significantly stronger in the presence of the BRAF/CRAF heterodimer. In cells, this complex is located at the membrane, which is to be expected as RAS is a membrane-bound protein. Nevertheless, the evidence suggests that membrane localization is not responsible for the dimerization of BRAF/CRAF. 14–3-3 is an additional protein necessary for the interaction between BRAF and CRAF. It is responsible for maintaining the complex structure and RAS-dependent RAF activation through the binding of CRAF’s S621 amino acid residue [92–94]. Although the three RAF isoforms are very similar, their ability to activate downstream targets is markedly different. As discussed above, the heterodimerization of different RAF isomers is important to their activity, with the most sensitive being CRAF’s dependence on the presence of BRAF. Likewise, BRAF’s activity is significantly lower in the absence of CRAF. ARAF, however, the least active isoform, is also the most dependent on BRAF but is independent of CRAF. It is of note that BRAF is capable of spontaneous homodimerization but that CRAF and ARAF are not. Mutations in *BRAF* and *CRAF* play significant roles by increasing homo and heterodimerization and catalytic activity [95]. Although ARAF is the least activate isoform of RAF, it is very significantly activated by the oncogenic Src protein [96].

### MEK1/2 and ERK1/2 regulation

The RAF protein kinase family is the major activator of the mitogen-activated protein kinases, MEK1 and MEK2 (MEK1/2) (Fig. 1). CRAF activates both MEK1 and MEK2, whereas ARAF and BRAF predominantly activate MEK1 [97]. To be fully active, MEK1/2 must be phosphorylated on two serine residues, at positions 218 and 222, located in the activation loop. Although RAF proteins are the predominant activators, MEK1/2 can also be phosphorylated at the same residues by MEK Kinase (MEKK), c-Mos, and growth factor-stimulated MEK1/2 activator [98–100]. Once activated, MEK1/2 can, in turn, activate its main substrates—ERK1 and ERK2—by phosphorylation. The ERK family is composed of several isoforms, from ERK1 to ERK8, but only ERK1 and ERK2 (also known as the p44 and p42 kinases, respectively) are involved in the RAS/MAPK signaling pathway. To learn more about other isoforms, see Bogoyevitch et al. [101]. In contrast to the activation of MEK1/2, no other ERK1/2 activators are currently known. MEK1/2 phosphorylates ERK1/2 at threonine 183 and tyrosine 185 in the Thr-Glu-Tyr motif [102]. ERK1/2 activation is facilitated by its spatial proximity to MEK1/2 within a protein complex including KSR and MP-1 [103]. The enzymatic reaction is enhanced by electrostatic interactions between the basic amino acids (lysine) present in the ERK2-docking site in the N-terminal region of MEK1 and the acidic amino acids (aspartate) located in the MEK1-docking site in the C-terminal portion of ERK2 [104, 105]. The specificity of the RAF-MEK1/2-ERK1/2 reactions is reinforced by the fact that MAPKs are proline-directed kinases, meaning that they strongly recognize their specific phosphorylation sites through the proline at position + 1 of them. This proline is required for efficient activation of MEK1/2 from RAF and of ERK1/2 from MEK1/2 [97, 106].

Upon activation, ERK dimerizes to form either ERK1 or ERK2 homodimers, although the functional implication of this dimerization remains unclear. Khokhlatchev et al. showed that this dimerization is involved in nuclear translocation, whereas most recently, Casar et al. demonstrated it to be essential for the activation of cytoplasmic targets [107, 108]. Via phosphorylation, active-ERK1/2 regulates a large number of cytoplasmic and nuclear targets which mainly regulate cell proliferation, differentiation, survival, and apoptosis (Figs. 1 and 2). Many of those targets contain a D domain and a DEF motif that improves the efficacy and specificity of the interaction with ERK1/2’s CD/CRS (common docking motif/cytosolic retention sequence) domain [109]. In 2017, based on various phospho-proteomic analyses, Ünal et al. reviewed 2507 ERK1/2-targets, of which 659 were direct and 1848 were indirect targets [110]. Direct targets are mainly involved in cell-cycle regulation and cell-signaling, for example, the well-known ERK1/2 targets, the Ets family of transcription factors—ELK-1, ELK-4, Ets-2—but also MYC, phospholipase A2, p90^RSK^, paxillin, and calnexin [67, 111–113]. Under stress conditions, ERK1/2 can become involved in cell death by phosphorylating p53 and thus inducing apoptosis [114]. However, ERK1/2 is also available for the activation of substrates in a phosphorylation-independent way through protein–protein interaction, such as with topoisomerase II alpha [115].

Importantly, the signal duration must be finely regulated to avoid the loss of cell control which can promote the development of diseases such as cancer. Negative feedback and phosphatase activity regulate the signal induced by the ERK1/2 / MAPK pathway. ERK1/2 can rapidly inhibit the pathway’s main components (e.g., MEK1/2, RAF, Sos1, RTK, KSR) through phosphorylation and regulate the expression of phosphatases such as dual-specificity MAPK phosphatase (MKPs) and Sprouty proteins, both of which inhibit the pathway. To learn more about this negative regulation, see the review by Lake et al. [116].

## RAS-MAPK inhibitors in clinics

There is thus strong evidence that the RAS-MAPK pathway plays an important role in the early development of NB, and this provides a rationale for the use of small-molecule inhibitors that can target the activated ALK and/or RAS-MAPK pathways. Many ongoing clinical trials testing the efficacy of RAS-MAPK pathway inhibitors against NB further underpin its importance. They are summarized in the Table 4 and visualized in Figs. 1 and 2.

**Table 4:** Active, recruiting, and completed phase I to III clinical trials for NB

Targeted protein	Inhibitor	Clinical Trial #	Phase	Combination
**ALK**	**Crizotinib**	**NCT00939770**	**I/II**	**None**
		**NCT03126916**	**III**	**Carboplatin / Cisplatin / Cyclophosphamide / Dinutuximab / Doxorubicin /** **Etoposide / Isotretinoin / Thiotepa / Topotecan / Radiation Therapy**
		**NCT01606878**	**I**	**Cyclophosphamide / Topotecan or Doxorubicin / Vincristine**
		**NCT03194893**	**III**	**None**
		**NCT01121588**	**I**	**None**
		**NTR5584**	**I**	**Temsirolimus**
	**Lorlatinib**	**NCT03107988**	**I**	**Cyclophosphamide / Topotecan**
	**Ceritinib**	**NCT02559778**	**II**	**DFMO**
		**NCT02780128**	**I**	**Ribociclib (CDK4/CDK6 inhibitor)**
		**NCT01742286**	**I**	**None**
	**Entrectinib**	**NCT02650401**^**a**^	**I/II**	**None**
	**Ensartinib**	**NCT03155620**	**II**	**None**
		**NCT03213652**	**II**	**None**
	**Alectinib**	**NCT03194893**	**III**	**None**
**FGFR**	**Erdafitinib**	**NCT03210714**	**II**	**None**
		**NCT03155620**	**II**	**None**
**RAS**	**Tipifarnib**	**NCT03155620**	**II**	**None**
		**NCT04284774**	**II**	**None**
**BRAF**	**Dabrafenib**	**NCT02124772**	**I/II**	**Trametinib**
	**Sorafenib**	**NCT02559778**	**II**	**DFMO**
		**NCT01518413**	**I**	**Irinotecan**
		**NCT01683149**	**I**	**Topotecan**
		**NCT00665990**	**I**	**Bevacizumab / Cyclophosphamide**
		**NCT02298348**	**I**	**Topotecan / Cyclophosphamide**
	**Vemurafenib**	**NCT03220035**	**II**	**None**
		**NCT01596140**	**I**	**Everolimus / Temsirolimus**
		**NCT03155620**	**II**	**None**
	**TAK-580**^**b**^	**NCT03429803**	**I**	**None**
**MEK 1/2**	**Trametinib**	**NCT03434262**	**I**	**Ribociclib**
		**NCT02124772**	**I/II**	**Dabrafenib**
	**Selumetinib**	**NCT03155620**	**II**	**None**
		**NCT03213691**	**II**	**None**
	**Cobimetinib**	**NCT02639546**	**I/II**	**None**
	**Binimetinib**	**NCT02285439**	**I/II**	**None**
**ERK 1/2**	**Ulixertinib**	**NCT03698994**	**II**	**None**
		**NCT03155620**	**II**	**None**

^a^Doebele RC, Drilon A, Paz-Ares L, Siena S, Shaw AT, Farago AF, Blakely CM, Seto T, Cho BC, Tosi D et al. Entrectinib in patients with advanced or metastatic NTRK fusion-positive solid tumours: integrated analysis of three phase 1–2 trials. Lancet Oncol. 2020; 21(2): 271–282

^b^pan-RAF inhibitor

The first phase I-II clinical trial testing an ALK inhibitor for NB was initiated more than 10 years ago; it evaluated crizotinib, which was first developed as a MET inhibitor [117]. Unfortunately, it was rapidly demonstrated that crizotinib’s ability to inhibit ALK activation was not constant and it was dependent on the type of *ALK* mutation and the crizotinib dose. Investigators also observed the emergence of resistance to this compound, leading to the development of new generations of ALK inhibitors such as alectinib, ceritinib, and lorlatinib. Table 5 summarizes the available data on how well ALK inhibitors inhibit different mutated ALKs. As they have tried to circumvent the emergence of resistance, due to the occurrence of either *ALK* mutations or downstream mutations in *PTPN11* and *NRAS*, researchers have focused on the downstream targets of the RAS-MAPK pathway.

**Table 5:** ALK mutations and their impact on the efficiency of ALK inhibitors

ALK mutation	Poorly effective	Effective	Highly effective	Reference
**G1123S**	Ceritinib			[118]
**G1128A**		Crizotinib, Ceritinib	Brigatinib, Lorlatinib, Alectinib	[119, 120]
**L1151Tins**	Crizotinib			[118]
**L1152R**	Crizotinib			[118]
**C1156Y**	Crizotinib			[118]
**I1171N**	Crizotinib	Ceritinib	Brigatinib, Lorlatinib, Alectinib	[118–120]
**I1171T**	Crizotinib, Alectinib	Ceritinib	Brigatinib, Lorlatinib, Alectinib	[119, 120]
**F1174C**	Ceritinib			[121]
**F1174L**	Crizotinib (partial inhibition)	Ceritinib	Brigatinib, Lorlatinib, Alectinib	[118–121]
**F1174V**	Crizotinib, Ceritinib			[118]
**R1192P**		Crizotinib, Ceritinib	Brigatinib, Lorlatinib, Alectinib	[119, 120]
**L1196M**	Crizotinib			[118]
**L1198F**	Lorlatinib			[118]
**G1202R**	Crizotinib, Ceritinib, Alectinib			[118]
**S1206Y**	Crizotinib			[118]
**F1245V**		Crizotinib, Ceritinib	Brigatinib, Lorlatinib	[119, 121]
**F1245C**	Crizotinib		Alectinib	[118, 120]
**G1269A**	Crizotinib	Ceritinib	Brigatinib, Lorlatinib, Alectinib	[118–120]
**R1275Q**		Crizotinib, Ceritinib	Brigatinib, Lorlatinib, Alectinib	[117, 119, 120]
**Y1278S**		Crizotinib, Ceritinib	Brigatinib, Lorlatinib, Alectinib	[119, 120]

Tipifarnib is the only RAS inhibitor currently being tested in clinical trials for NB. It is a general inhibitor of RAS proteins, through the inhibition of the RAS farnesylation that prevents RAS localization to cellular membranes, thereby inhibiting signaling [122]. Activated RAS’ first downstream target is BRAF. Kakodkar et al. demonstrated that BRAF inhibition using Sorafenib inhibited NB growth in vivo, attenuated ERK signaling, and arrested the cell cycle at the G1/G0 stage [123]. Interestingly, Sorafenib showed a transient anti-tumor effect and short temporal stabilization in four NB patients treated with it on a companionate use basis [124]; it is currently being investigated in five early-stage clinical trials. BRAF is also a target of TAK-580, a pan-RAF inhibitor, presently being tested in a phase I study.

More recently, MEK1/2 inhibitors such as trametinib, cobimetinib, binimetinib, and selumetinib were shown to inhibit the growth of different NB cell lines harboring RAS-MAPK mutations [125–127]. Interestingly, sensitivity to MEK1/2 inhibition was found to be correlated with the disruption of specific genes. Cell lines with *RAS/BRAF* mutations were found to be more sensitive than cells with *NF1* deletion, whereas cells with *ALK* mutations, which consistently showed some sensitivity to MEK1/2 inhibitors, were the least susceptible to MEK1/2 inhibitors [16]. Interestingly some studies even reported that MEK1/2 inhibition was not always effective against NB cell lines [125–127], particularly cell lines presenting *ALK* addiction. In these cell lines, trametinib was found to increase the activation of AKT and ERK5 kinase, leading to increased cell proliferation and survival [125].

Different studies have reported that the sensitivity of NB cells to MEK1/2 inhibitors was positively correlated with their endogenous levels of phosphorylated ERK1/2 (pERK1/2) [126, 128]. In response to binimetinib, sensitive cells demonstrated a complete reduction of pERK1/2, whereas the resistant ones demonstrated incomplete reduction or retention of pERK1/2. Furthermore, basal NF1 protein expression was also found to be correlated with binimetinib response, with reduced expression being associated with higher sensitivity to MEK inhibition. These results suggest that the expression of pERK1/2 and NF1 could be used as biomarkers helping to identify a patient who may benefit from treatment with binimetinib [126]. Unfortunately, recent results suggest that long-term exposure to MAPK pathway inhibition could result in acquired resistance, by relieving the ERK1/2-dependent feedback inhibition of RAF and the reactivation of the MAPK pathway [128, 129].

To overcome the development of resistance through the occurrence of downstream activation mutations, the simultaneous inhibition of the RAS-MAPK pathway at several points has been proposed for NB. Valencia-Sama et al. investigated combinations of SHP2 inhibitors and inhibitors of RAF, MEK1/2, and ERK1/2. These researchers demonstrated the synergistic effects in vitro and in vivo of combining SHP2 with BRAF (vemurafenib), MEK1/2 (trametinib), or ERK1/2 (ulixertinib) inhibition in RAS-activating tumors (NRAS^Q61K^) that were resistant to SHP2 inhibitors alone [130]. Concurrent inhibition using MEK1/2 (trametinib) and BRAF (dabrafenib) is currently being evaluated in a phase I/II clinical trial.

The RAS-MAPK pathway regulates many different cellular processes, as well as crosstalk with the PI3K/AKT/mTOR pathway (Fig. 2). It has been proposed that inhibition of the RAS-MAPK pathway should be supplemented with inhibition of other cellular functions, either to improve the efficacy of RAS-MAPK inhibitors or to help avoid the development of resistance.

PI3K/AKT/mTOR is one of the main signaling pathways that is frequently dysregulated in many different cancers, including NB [131]. This pathway shares many upstream activators and downstream targets with the RAS-MAPK pathway, and the crosstalk between them first attracted the attention of researchers in the early 1990s. It was demonstrated that pathways could interact either through mutual inhibition or activation and that they were instrumental in the progressive adaptations to activating RAS-MAPK mutations, which cause significant oncogenic stress [131]. In addition, many well documented examples have demonstrated the importance of both pathways in the development of resistance to inhibitors of either pathway [132]. The simultaneous inhibition of RAS-MAPK and PI3K/AKT/mTOR in in vitro and in vivo NB models has demonstrated the synergistic effects between crizotinib and Torin2 (mTOR inhibitor) [133, 134] and has recently resulted in the opening of an early stage clinical trial to test the toxicity of a combined ALK and PI3K/AKT/mTOR inhibitors treatment.

As discussed above, Cyclin D, which is regulated by ERK1/2, is an important oncogene that promotes transition from the G1 phase to the S phase of the cell cycle. A preclinical study of Cyclin D inhibition, using a CDK4/6 inhibitor (LEEO11) on a panel of NB cell lines, demonstrated its potential for application against NB [135]. In a recent study, Wood et al. demonstrated a synergy between ceritinib and ribociclib, another inhibitor of CDK4/6 [136]. Later, Hart et al. obtained similar results using the MEK1/2 inhibitors binimetinib and ribociclib [125, 127]. The NEPENTHE study is currently assessing the feasibility of using ceritinib and ribociclib in combination for the treatment of NB.

Other interesting combinations have included inhibition of ALK and MDM2, which demonstrated that it was able to overcome the resistance to ceritinib conferred by MYCN upregulation [137]. Inhibition of YAP1 (hippo signaling) resulted in RAS-driven NB cells being sensitized to trametinib MEK1/2 and in enhanced G1/S cell-cycle arrest [138]. Regorafenib, which is known to inhibit the RAS/MAPK, PI3K/Akt/mTOR, and Fos/Jun pathways, has also shown good in vivo and in vitro efficacy against NB cells [139].

## Conclusions

Even though NB is very well-known as a cancer with a low frequency of point mutations, the RAS-MAPK pathway shows a high degree of dysregulation. Since mutations in genes related to the RAS-MAPK pathway could lead to its dysregulation, looking at the frequency of directly activated mutations probably underestimates the actual frequency of dysfunctional RAS-MAPK in NB. Although this pathway seems to offer many targets for inhibition, many years of clinical investigation have proved that inhibition is a challenging task due to the frequent development of resistance, complex feedback-loop regulation, and a large number of interactions with other signaling pathways that can affect the functioning of the RAS-MAPK pathway. This last aspect is a particularly important task, and greater efforts should be invested in understanding how the RAS-MAPK pathway functions in the molecular background of NB cells. Current evidence suggests that inhibition of the RAS-MAPK pathway alone will not be sufficient to adequately target NB. Rather, it is becoming apparent that the inhibition of other pathways, such as PI3K/AKT/mTOR, or processes like the cell cycle, will be vital to achieving effective treatments for NB and avoiding the development of resistance.

## Supplementary Information

**Additional file 1.**

## Data Availability

Not applicable.

## Abbreviations

CD: Common docking
COSMIC: Catalogue of somatic mutations in cancer
CRS: Cytosolic retention sequence
INRG: International Neuroblastoma Risk Group
IPA: Ingenuity Pathway Analysis software
MNA: MYCN amplification
NB: Neuroblastoma
RTK: Receptor tyrosine kinase
SNV: Single nucleotide variants

## References

[1] Maris JM, Hogarty MD, Bagatell R, Cohn SL (2007). Neuroblastoma. Lancet.

[2] Park JR, Eggert A, Caron H (2008). Neuroblastoma: biology, prognosis, and treatment. Pediatr Clin N Am.

[3] Brodeur GM (2003). Neuroblastoma: biological insights into a clinical enigma. Nat Rev Cancer.

[4] Pastor ER, Mousa SA (2019). Current management of neuroblastoma and future direction. Crit Rev Oncol Hematol.

[5] Maris JM (2010). Recent advances in neuroblastoma. N Engl J Med.

[6] Matthay KK, Maris JM, Schleiermacher G, Nakagawara A, Mackall CL, Diller L, Weiss WA (2016). Neuroblastoma. Nat Rev Dis Primers.

[7] Mlakar V, Jurkovic Mlakar S, Lopez G, Maris JM, Ansari M, Gumy-Pause F (2017). 11q deletion in neuroblastoma: a review of biological and clinical implications. Mol Cancer.

[8] Cohn SL, Pearson AD, London WB, Monclair T, Ambros PF, Brodeur GM, Faldum A, Hero B, Iehara T, Machin D (2009). The International Neuroblastoma Risk Group (INRG) classification system: an INRG Task Force report. J Clin Oncol.

[9] Mosse YP, Laudenslager M, Longo L, Cole KA, Wood A, Attiyeh EF, Laquaglia MJ, Sennett R, Lynch JE, Perri P (2008). Identification of ALK as a major familial neuroblastoma predisposition gene. Nature.

[10] Janoueix-Lerosey I, Lequin D, Brugieres L, Ribeiro A, de Pontual L, Combaret V, Raynal V, Puisieux A, Schleiermacher G, Pierron G (2008). Somatic and germline activating mutations of the ALK kinase receptor in neuroblastoma. Nature.

[11] Chen Y, Takita J, Choi YL, Kato M, Ohira M, Sanada M, Wang L, Soda M, Kikuchi A, Igarashi T (2008). Oncogenic mutations of ALK kinase in neuroblastoma. Nature.

[12] Caren H, Abel F, Kogner P, Martinsson T (2008). High incidence of DNA mutations and gene amplifications of the ALK gene in advanced sporadic neuroblastoma tumours. Biochem J.

[13] George RE, Sanda T, Hanna M, Frohling S, Luther W, Zhang J, Ahn Y, Zhou W, London WB, McGrady P (2008). Activating mutations in ALK provide a therapeutic target in neuroblastoma. Nature.

[14] Pugh TJ, Morozova O, Attiyeh EF, Asgharzadeh S, Wei JS, Auclair D, Carter SL, Cibulskis K, Hanna M, Kiezun A (2013). The genetic landscape of high-risk neuroblastoma. Nat Genet.

[15] Bresler SC, Weiser DA, Huwe PJ, Park JH, Krytska K, Ryles H, Laudenslager M, Rappaport EF, Wood AC, McGrady PW (2014). ALK mutations confer differential oncogenic activation and sensitivity to ALK inhibition therapy in neuroblastoma. Cancer Cell.

[16] Eleveld TF, Oldridge DA, Bernard V, Koster J, Colmet Daage L, Diskin SJ, Schild L, Bentahar NB, Bellini A, Chicard M (2015). Relapsed neuroblastomas show frequent RAS-MAPK pathway mutations. Nat Genet.

[17] Tolbert VP, Coggins GE, Maris JM (2017). Genetic susceptibility to neuroblastoma. Curr Opin Genet Dev.

[18] Tonini GP, Capasso M (2020). Genetic predisposition and chromosome instability in neuroblastoma. Cancer Metastasis Rev.

[19] Osajima-Hakomori Y, Miyake I, Ohira M, Nakagawara A, Nakagawa A, Sakai R (2005). Biological role of anaplastic lymphoma kinase in neuroblastoma. Am J Pathol.

[20] Miyake I, Hakomori Y, Shinohara A, Gamou T, Saito M, Iwamatsu A, Sakai R (2002). Activation of anaplastic lymphoma kinase is responsible for hyperphosphorylation of ShcC in neuroblastoma cell lines. Oncogene.

[21] Ma X, Liu Y, Liu Y, Alexandrov LB, Edmonson MN, Gawad C, Zhou X, Li Y, Rusch MC, Easton J, Huether R, Gonzalez-Pena V, Wilkinson MR, Hermida LC, Davis S, Sioson E, Pounds S, Cao X, Ries RE, Wang Z, Chen X, Dong L, Diskin SJ, Smith MA, Guidry Auvil JM, Meltzer PS, Lau CC, Perlman EJ, Maris JM, Meshinchi S, Hunger SP, Gerhard DS, Zhang J (2018). Pan-cancer genome and transcriptome analyses of 1,699 paediatric leukaemias and solid tumours. Nature.

[22] De Brouwer S, De Preter K, Kumps C, Zabrocki P, Porcu M, Westerhout EM, Lakeman A, Vandesompele J, Hoebeeck J, Van Maerken T (2010). Meta-analysis of neuroblastomas reveals a skewed ALK mutation spectrum in tumors with MYCN amplification. Clin Cancer Res.

[23] Trigg RM, Turner SD. ALK in neuroblastoma: biological and therapeutic implications. Cancers (Basel). 2018;10. 10.3390/cancers10040113. 10.3390/cancers10040113 PMC5923368 29642598

[24] Padovan-Merhar OM, Raman P, Ostrovnaya I, Kalletla K, Rubnitz KR, Sanford EM, Ali SM, Miller VA, Mosse YP, Granger MP (2016). Enrichment of targetable mutations in the relapsed neuroblastoma genome. PLoS Genet.

[25] Ackermann S, Cartolano M, Hero B, Welte A, Kahlert Y, Roderwieser A, Bartenhagen C, Walter E, Gecht J, Kerschke L (2018). A mechanistic classification of clinical phenotypes in neuroblastoma. Science.

[26] Shukla N, Ameur N, Yilmaz I, Nafa K, Lau CY, Marchetti A, Borsu L, Barr FG, Ladanyi M (2012). Oncogene mutation profiling of pediatric solid tumors reveals significant subsets of embryonal rhabdomyosarcoma and neuroblastoma with mutated genes in growth signaling pathways. Clin Cancer Res.

[27] Ireland CM (1989). Activated N-ras oncogenes in human neuroblastoma. Cancer Res.

[28] Moley JF, Brother MB, Wells SA, Spengler BA, Biedler JL, Brodeur GM (1991). Low frequency of ras gene mutations in neuroblastomas, pheochromocytomas, and medullary thyroid cancers. Cancer Res.

[29] Schubbert S, Shannon K, Bollag G (2007). Hyperactive Ras in developmental disorders and cancer. Nat Rev Cancer.

[30] Eleveld TF, Schild L, Koster J, Zwijnenburg DA, Alles LK, Ebus ME, Volckmann R, Tijtgat GA, van Sluis P, Versteeg R, Molenaar JJ (2018). RAS-MAPK pathway-driven tumor progression is associated with loss of CIC and other genomic aberrations in neuroblastoma. Cancer Res.

[31] Li H, Yu Y, Zhao Y, Wu D, Yu X, Lu J, Chen Z, Zhang H, Hu Y, Zhai Y, Su J, Aheman A, de las Casas A, Jin J, Xu X, Shi Z, Woodfield SE, Vasudevan SA, Agarwal S, Yan Y, Yang J, Foster JH (2019). Small molecule inhibitor agerafenib effectively suppresses neuroblastoma tumor growth in mouse models via inhibiting ERK MAPK signaling. Cancer Lett.

[32] Davies H, Bignell GR, Cox C, Stephens P, Edkins S, Clegg S, Teague J, Woffendin H, Garnett MJ, Bottomley W (2002). Mutations of the BRAF gene in human cancer. Nature.

[33] Marks JL, Gong Y, Chitale D, Golas B, McLellan MD, Kasai Y, Ding L, Mardis ER, Wilson RK, Solit D (2008). Novel MEK1 mutation identified by mutational analysis of epidermal growth factor receptor signaling pathway genes in lung adenocarcinoma. Cancer Res.

[34] Aurtenetxe O, Zaldumbide L, Erramuzpe A, Lopez R, Lopez JI, Cortes JM, Pulido R, Nunes-Xavier CE (2018). DUSP5 expression associates with poor prognosis in human neuroblastoma. Exp Mol Pathol.

[35] Esposito MR, Binatti A, Pantile M, Coppe A, Mazzocco K, Longo L, Capasso M, Lasorsa VA, Luksch R, Bortoluzzi S, Tonini GP (2018). Somatic mutations in specific and connected subpathways are associated with short neuroblastoma patients’ survival and indicate proteins targetable at onset of disease. Int J Cancer.

[36] Schleiermacher G, Javanmardi N, Bernard V, Leroy Q, Cappo J, Rio Frio T, Pierron G, Lapouble E, Combaret V, Speleman F, de Wilde B, Djos A, Øra I, Hedborg F, Träger C, Holmqvist BM, Abrahamsson J, Peuchmaur M, Michon J, Janoueix-Lerosey I, Kogner P, Delattre O, Martinsson T (2014). Emergence of new ALK mutations at relapse of neuroblastoma. J Clin Oncol.

[37] Der CJ, Finkel T, Cooper GM (1986). Biological and biochemical properties of human rasH genes mutated at codon 61. Cell.

[38] Trochet D, Bourdeaut F, Janoueix-Lerosey I, Deville A, de Pontual L, Schleiermacher G, Coze C, Philip N, Frebourg T, Munnich A (2004). Germline mutations of the paired-like homeobox 2B (PHOX2B) gene in neuroblastoma. Am J Hum Genet.

[39] Mosse YP, Laudenslager M, Khazi D, Carlisle AJ, Winter CL, Rappaport E, Maris JM (2004). Germline PHOX2B mutation in hereditary neuroblastoma. Am J Hum Genet.

[40] Devoto M, Specchia C, Laudenslager M, Longo L, Hakonarson H, Maris J, Mosse Y (2011). Genome-wide linkage analysis to identify genetic modifiers of ALK mutation penetrance in familial neuroblastoma. Hum Hered.

[41] Kamihara J, Bourdeaut F, Foulkes WD, Molenaar JJ, Mosse YP, Nakagawara A, Parareda A, Scollon SR, Schneider KW, Skalet AH (2017). Retinoblastoma and neuroblastoma predisposition and surveillance. Clin Cancer Res.

[42] Seidinger AL, Fortes FP, Mastellaro MJ, Cardinalli IA, Zambaldi LG, Aguiar SS, Yunes JA (2015). Occurrence of neuroblastoma among TP53 p.R337H carriers. PLoS One.

[43] Schimke RN, Collins DL, Stolle CA (2010). Paraganglioma, neuroblastoma, and a SDHB mutation: resolution of a 30-year-old mystery. Am J Med Genet A.

[44] Maas SM, Vansenne F, Kadouch DJ, Ibrahim A, Bliek J, Hopman S, Mannens MM, Merks JH, Maher ER, Hennekam RC (2016). Phenotype, cancer risk, and surveillance in Beckwith-Wiedemann syndrome depending on molecular genetic subgroups. Am J Med Genet A.

[45] Kratz CP, Rapisuwon S, Reed H, Hasle H, Rosenberg PS (2011). Cancer in Noonan, Costello, cardiofaciocutaneous and LEOPARD syndromes. Am J Med Genet C Semin Med Genet.

[46] Brems H, Beert E, de Ravel T, Legius E (2009). Mechanisms in the pathogenesis of malignant tumours in neurofibromatosis type 1. Lancet Oncol.

[47] Rybinski B, Wolinsky T, Brohl A, Moerdler S, Reed DR, Ewart M, Weiser D (2020). Multifocal primary neuroblastoma tumor heterogeneity in siblings with co-occurring PHOX2B and NF1 genetic aberrations. Genes Chromosomes Cancer.

[48] Jones SM, Kazlauskas A (2001). Growth-factor-dependent mitogenesis requires two distinct phases of signalling. Nat Cell Biol.

[49] Vasjari L, Bresan S, Biskup C, Pai G, Rubio I (2019). Ras signals principally via Erk in G1 but cooperates with PI3K/Akt for Cyclin D induction and S-phase entry. Cell Cycle.

[50] Brady SW, Liu Y, Ma X, Gout AM, Hagiwara K, Zhou X, Wang J, Macias M, Chen X, Easton J, Mulder HL, Rusch M, Wang L, Nakitandwe J, Lei S, Davis EM, Naranjo A, Cheng C, Maris JM, Downing JR, Cheung NKV, Hogarty MD, Dyer MA, Zhang J (2020). Pan-neuroblastoma analysis reveals age- and signature-associated driver alterations. Nat Commun.

[51] Edlich F (2018). BCL-2 proteins and apoptosis: recent insights and unknowns. Biochem Biophys Res Commun.

[52] Yang JY, Zong CS, Xia W, Yamaguchi H, Ding Q, Xie X, Lang JY, Lai CC, Chang CJ, Huang WC (2008). ERK promotes tumorigenesis by inhibiting FOXO3a via MDM2-mediated degradation. Nat Cell Biol.

[53] Ying H, Kimmelman AC, Lyssiotis CA, Hua S, Chu GC, Fletcher-Sananikone E, Locasale JW, Son J, Zhang H, Coloff JL (2012). Oncogenic Kras maintains pancreatic tumors through regulation of anabolic glucose metabolism. Cell.

[54] Hall A, Meyle KD, Lange MK, Klima M, Sanderhoff M, Dahl C, Abildgaard C, Thorup K, Moghimi SM, Jensen PB (2013). Dysfunctional oxidative phosphorylation makes malignant melanoma cells addicted to glycolysis driven by the (V600E)BRAF oncogene. Oncotarget.

[55] Falck Miniotis M, Arunan V, Eykyn TR, Marais R, Workman P, Leach MO, Beloueche-Babari M (2013). MEK1/2 inhibition decreases lactate in BRAF-driven human cancer cells. Cancer Res.

[56] Kim JW, Gao P, Liu YC, Semenza GL, Dang CV (2007). Hypoxia-inducible factor 1 and dysregulated c-Myc cooperatively induce vascular endothelial growth factor and metabolic switches hexokinase 2 and pyruvate dehydrogenase kinase 1. Mol Cell Biol.

[57] Parmenter TJ, Kleinschmidt M, Kinross KM, Bond ST, Li J, Kaadige MR, Rao A, Sheppard KE, Hugo W, Pupo GM (2014). Response of BRAF-mutant melanoma to BRAF inhibition is mediated by a network of transcriptional regulators of glycolysis. Cancer Discov.

[58] Shim H, Dolde C, Lewis BC, Wu CS, Dang G, Jungmann RA, Dalla-Favera R, Dang CV (1997). c-Myc transactivation of LDH-A: implications for tumor metabolism and growth. Proc Natl Acad Sci U S A.

[59] Ng HH, Surani MA (2011). The transcriptional and signalling networks of pluripotency. Nat Cell Biol.

[60] Chung J, Uchida E, Grammer TC, Blenis J (1997). STAT3 serine phosphorylation by ERK-dependent and -independent pathways negatively modulates its tyrosine phosphorylation. Mol Cell Biol.

[61] Sengupta TK, Talbot ES, Scherle PA, Ivashkiv LB (1998). Rapid inhibition of interleukin-6 signaling and Stat3 activation mediated by mitogen-activated protein kinases. Proc Natl Acad Sci U S A.

[62] Ueda T, Watanabe-Fukunaga R, Fukuyama H, Nagata S, Fukunaga R (2004). Mnk2 and Mnk1 are essential for constitutive and inducible phosphorylation of eukaryotic initiation factor 4E but not for cell growth or development. Mol Cell Biol.

[63] Pelletier J, Graff J, Ruggero D, Sonenberg N (2015). Targeting the eIF4F translation initiation complex: a critical nexus for cancer development. Cancer Res.

[64] Lavoie H, Gagnon J, Therrien M (2020). ERK signalling: a master regulator of cell behaviour, life and fate. Nat Rev Mol Cell Biol.

[65] Hubner A, Barrett T, Flavell RA, Davis RJ (2008). Multisite phosphorylation regulates Bim stability and apoptotic activity. Mol Cell.

[66] Kennedy D, Mnich K, Oommen D, Chakravarthy R, Almeida-Souza L, Krols M, Saveljeva S, Doyle K, Gupta S, Timmerman V, Janssens S, Gorman AM, Samali A (2017). HSPB1 facilitates ERK-mediated phosphorylation and degradation of BIM to attenuate endoplasmic reticulum stress-induced apoptosis. Cell Death Dis.

[67] Bonni A, Brunet A, West AE, Datta SR, Takasu MA, Greenberg ME (1999). Cell survival promoted by the Ras-MAPK signaling pathway by transcription-dependent and -independent mechanisms. Science.

[68] Mendoza MC, Er EE, Zhang W, Ballif BA, Elliott HL, Danuser G, Blenis J (2011). ERK-MAPK drives lamellipodia protrusion by activating the WAVE2 regulatory complex. Mol Cell.

[69] Miki H, Fukuda M, Nishida E, Takenawa T (1999). Phosphorylation of WAVE downstream of mitogen-activated protein kinase signaling. J Biol Chem.

[70] Choi C, Helfman DM (2014). The Ras-ERK pathway modulates cytoskeleton organization, cell motility and lung metastasis signature genes in MDA-MB-231 LM2. Oncogene.

[71] Drosten M, Dhawahir A, Sum EY, Urosevic J, Lechuga CG, Esteban LM, Castellano E, Guerra C, Santos E, Barbacid M (2010). Genetic analysis of Ras signalling pathways in cell proliferation, migration and survival. EMBO J.

[72] Klein RM, Spofford LS, Abel EV, Ortiz A, Aplin AE (2008). B-RAF regulation of Rnd3 participates in actin cytoskeletal and focal adhesion organization. Mol Biol Cell.

[73] Sugimoto T, Kuroda H, Horii Y, Moritake H, Tanaka T, Hattori S (2001). Signal transduction pathways through TRK-A and TRK-B receptors in human neuroblastoma cells. Jpn J Cancer Res.

[74] Jones DT, Hutter B, Jager N, Korshunov A, Kool M, Warnatz HJ, Zichner T, Lambert SR, Ryzhova M, Quang DA (2013). Recurrent somatic alterations of FGFR1 and NTRK2 in pilocytic astrocytoma. Nat Genet.

[75] Lemmon MA, Schlessinger J (2010). Cell signaling by receptor tyrosine kinases. Cell..

[76] Pacenta HL, Macy ME (2018). Entrectinib and other ALK/TRK inhibitors for the treatment of neuroblastoma. Drug Des Devel Ther.

[77] Prigent SA, Nagane M, Lin H, Huvar I, Boss GR, Feramisco JR, Cavenee WK, Huang HS (1996). Enhanced tumorigenic behavior of glioblastoma cells expressing a truncated epidermal growth factor receptor is mediated through the Ras-Shc-Grb2 pathway. J Biol Chem.

[78] Vogel W, Ullrich A (1996). Multiple in vivo phosphorylated tyrosine phosphatase SHP-2 engages binding to Grb2 via tyrosine 584. Cell Growth Differ.

[79] Zhang J, Zhang F, Niu R (2015). Functions of Shp2 in cancer. J Cell Mol Med.

[80] Chan G, Kalaitzidis D, Neel BG (2008). The tyrosine phosphatase Shp2 (PTPN11) in cancer. Cancer Metastasis Rev.

[81] Salcini AE, McGlade J, Pelicci G, Nicoletti I, Pawson T, Pelicci PG (1994). Formation of Shc-Grb2 complexes is necessary to induce neoplastic transformation by overexpression of Shc proteins. Oncogene.

[82] Bentires-Alj M, Paez JG, David FS, Keilhack H, Halmos B, Naoki K, Maris JM, Richardson A, Bardelli A, Sugarbaker DJ (2004). Activating mutations of the noonan syndrome-associated SHP2/PTPN11 gene in human solid tumors and adult acute myelogenous leukemia. Cancer Res.

[83] Rojas JM, Subleski M, Coque JJ, Guerrero C, Saez R, Li BQ, Lopez E, Zarich N, Aroca P, Kamata T (1999). Isoform-specific insertion near the Grb2-binding domain modulates the intrinsic guanine nucleotide exchange activity of hSos1. Oncogene.

[84] Overbeck AF, Brtva TR, Cox AD, Graham SM, Huff SY, Khosravi-Far R, Quilliam LA, Solski PA, Der CJ (1995). Guanine nucleotide exchange factors: activators of Ras superfamily proteins. Mol Reprod Dev.

[85] Kholodenko BN (2003). Four-dimensional organization of protein kinase signaling cascades: the roles of diffusion, endocytosis and molecular motors. J Exp Biol.

[86] Jiang X, Sorkin A (2002). Coordinated traffic of Grb2 and Ras during epidermal growth factor receptor endocytosis visualized in living cells. Mol Biol Cell.

[87] Cichowski K, Santiago S, Jardim M, Johnson BW, Jacks T (2003). Dynamic regulation of the Ras pathway via proteolysis of the NF1 tumor suppressor. Genes Dev.

[88] Holzel M, Huang S, Koster J, Ora I, Lakeman A, Caron H, Nijkamp W, Xie J, Callens T, Asgharzadeh S (2010). NF1 is a tumor suppressor in neuroblastoma that determines retinoic acid response and disease outcome. Cell.

[89] Buday L, Warne PH, Downward J (1995). Downregulation of the Ras activation pathway by MAP kinase phosphorylation of Sos. Oncogene.

[90] Jaitner BK, Becker J, Linnemann T, Herrmann C, Wittinghofer A, Block C (1997). Discrimination of amino acids mediating Ras binding from noninteracting residues affecting raf activation by double mutant analysis. J Biol Chem.

[91] Weber CK, Slupsky JR, Kalmes HA, Rapp UR (2001). Cancer Res.

[92] Luo ZJ, Zhang XF, Rapp U, Avruch J (1995). Identification of the 14.3.3 zeta domains important for self-association and Raf binding. J Biol Chem.

[93] Roy S, McPherson RA, Apolloni A, Yan J, Lane A, Clyde-Smith J, Hancock JF (1998). 14-3-3 facilitates Ras-dependent Raf-1 activation in vitro and in vivo. Mol Cell Biol.

[94] Tzivion G, Luo Z, Avruch J (1998). A dimeric 14-3-3 protein is an essential cofactor for Raf kinase activity. Nature.

[95] Freeman AK, Ritt DA, Morrison DK (2013). Effects of Raf dimerization and its inhibition on normal and disease-associated Raf signaling. Mol Cell.

[96] Marais R, Light Y, Paterson HF, Mason CS, Marshall CJ (1997). Differential regulation of Raf-1, A-Raf, and B-Raf by oncogenic ras and tyrosine kinases. J Biol Chem.

[97] Schaeffer HJ, Weber MJ (1999). Mitogen-activated protein kinases: specific messages from ubiquitous messengers. Mol Cell Biol.

[98] Lange-Carter CA, Pleiman CM, Gardner AM, Blumer KJ, Johnson GL (1993). A divergence in the MAP kinase regulatory network defined by MEK kinase and Raf. Science.

[99] Posada J, Yew N, Ahn NG, Vande Woude GF, Cooper JA (1993). Mos stimulates MAP kinase in Xenopus oocytes and activates a MAP kinase kinase in vitro. Mol Cell Biol.

[100] Zheng CF, Ohmichi M, Saltiel AR, Guan KL (1994). Growth factor induced MEK activation is primarily mediated by an activator different from c-raf. Biochemistry.

[101] Bogoyevitch MA, Court NW (2004). Counting on mitogen-activated protein kinases--ERKs 3, 4, 5, 6, 7 and 8. Cell Signal.

[102] Kolch W (2000). Meaningful relationships: the regulation of the Ras/Raf/MEK/ERK pathway by protein interactions. Biochem J.

[103] Ritt DA, Daar IO, Morrison DK (2006). KSR regulation of the Raf-MEK-ERK cascade. Methods Enzymol.

[104] Fukuda M, Gotoh Y, Nishida E (1997). Interaction of MAP kinase with MAP kinase kinase: its possible role in the control of nucleocytoplasmic transport of MAP kinase. EMBO J.

[105] Lange DE, Rager H, Plagmann HC, Baumann M (1976). Studies on the effectiveness of water spray devices in the gingival region. Dtsch Zahnarztl Z.

[106] Catling AD, Schaeffer HJ, Reuter CW, Reddy GR, Weber MJ (1995). A proline-rich sequence unique to MEK1 and MEK2 is required for raf binding and regulates MEK function. Mol Cell Biol.

[107] Casar B, Pinto A, Crespo P (2008). Essential role of ERK dimers in the activation of cytoplasmic but not nuclear substrates by ERK-scaffold complexes. Mol Cell.

[108] Khokhlatchev AV, Canagarajah B, Wilsbacher J, Robinson M, Atkinson M, Goldsmith E, Cobb MH (1998). Phosphorylation of the MAP kinase ERK2 promotes its homodimerization and nuclear translocation. Cell.

[109] Yoon S, Seger R (2006). The extracellular signal-regulated kinase: multiple substrates regulate diverse cellular functions. Growth Factors.

[110] Unal EB, Uhlitz F, Bluthgen N (2017). A compendium of ERK targets. FEBS Lett.

[111] Chevet E, Wong HN, Gerber D, Cochet C, Fazel A, Cameron PH, Gushue JN, Thomas DY, Bergeron JJ (1999). Phosphorylation by CK2 and MAPK enhances calnexin association with ribosomes. EMBO J.

[112] Roberts PJ, Der CJ (2007). Targeting the Raf-MEK-ERK mitogen-activated protein kinase cascade for the treatment of cancer. Oncogene.

[113] Sa G, Murugesan G, Jaye M, Ivashchenko Y, Fox PL (1995). Activation of cytosolic phospholipase A2 by basic fibroblast growth factor via a p42 mitogen-activated protein kinase-dependent phosphorylation pathway in endothelial cells. J Biol Chem.

[114] She QB, Chen N, Dong Z (2000). ERKs and p38 kinase phosphorylate p53 protein at serine 15 in response to UV radiation. J Biol Chem.

[115] Shapiro PS, Whalen AM, Tolwinski NS, Wilsbacher J, Froelich-Ammon SJ, Garcia M, Osheroff N, Ahn NG (1999). Extracellular signal-regulated kinase activates topoisomerase IIalpha through a mechanism independent of phosphorylation. Mol Cell Biol.

[116] Lake D, Correa SA, Muller J (2016). Negative feedback regulation of the ERK1/2 MAPK pathway. Cell Mol Life Sci.

[117] Mosse YP, Lim MS, Voss SD, Wilner K, Ruffner K, Laliberte J, Rolland D, Balis FM, Maris JM, Weigel BJ (2013). Safety and activity of crizotinib for paediatric patients with refractory solid tumours or anaplastic large-cell lymphoma: a Children’s Oncology Group phase 1 consortium study. Lancet Oncol.

[118] Hallberg B, Palmer RH (2016). The role of the ALK receptor in cancer biology. Ann Oncol.

[119] Umapathy G, Mendoza-Garcia P, Hallberg B, Palmer RH (2019). Targeting anaplastic lymphoma kinase in neuroblastoma. APMIS.

[120] Alam MW, Borenäs M, Lind DE, Cervantes-Madrid D, Umapathy G, Palmer RH, Hallberg B (2019). Alectinib, an anaplastic lymphoma kinase inhibitor, abolishes ALK activity and growth in ALK-positive neuroblastoma cells. Front Oncol.

[121] Bresler SC, Wood AC, Haglund EA, Courtright J, Belcastro LT, Plegaria JS, Cole K, Toporovskaya Y, Zhao H, Carpenter EL (2011). Differential inhibitor sensitivity of anaplastic lymphoma kinase variants found in neuroblastoma. Sci Transl Med.

[122] Cox AD, Der CJ, Philips MR (2015). Targeting RAS membrane association: back to the future for anti-RAS drug discovery?. Clin Cancer Res.

[123] Kakodkar NC, Peddinti RR, Tian Y, Guerrero LJ, Chlenski A, Yang Q, Salwen HR, Maitland ML, Cohn SL (2012). Sorafenib inhibits neuroblastoma cell proliferation and signaling, blocks angiogenesis, and impairs tumor growth. Pediatr Blood Cancer.

[124] Okada K, Nakano Y, Yamasaki K, Nitani C, Fujisaki H, Hara J (2016). Sorafenib treatment in children with relapsed and refractory neuroblastoma: an experience of four cases. Cancer Med.

[125] Umapathy G, Guan J, Gustafsson DE, Javanmardi N, Cervantes-Madrid D, Djos A, et al. MEK inhibitor trametinib does not prevent the growth of anaplastic lymphoma kinase (ALK)-addicted neuroblastomas. Sci Signal. 2017;10. 10.1126/scisignal.aam7550. 10.1126/scisignal.aam7550 29184034

[126] Woodfield SE, Zhang L, Scorsone KA, Liu Y, Zage PE (2016). Binimetinib inhibits MEK and is effective against neuroblastoma tumor cells with low NF1 expression. BMC Cancer.

[127] Hart LS, Rader J, Raman P, Batra V, Russell MR, Tsang M, Gagliardi M, Chen L, Martinez D, Li Y, Wood A, Kim S, Parasuraman S, Delach S, Cole KA, Krupa S, Boehm M, Peters M, Caponigro G, Maris JM (2017). Preclinical therapeutic synergy of MEK1/2 and CDK4/6 inhibition in neuroblastoma. Clin Cancer Res.

[128] Takeuchi Y, Tanaka T, Higashi M, Fumino S, Iehara T, Hosoi H, Sakai T, Tajiri T (2018). In vivo effects of short- and long-term MAPK pathway inhibition against neuroblastoma. J Pediatr Surg.

[129] Ishii N, Harada N, Joseph EW, Ohara K, Miura T, Sakamoto H, Matsuda Y, Tomii Y, Tachibana-Kondo Y, Iikura H, Aoki T, Shimma N, Arisawa M, Sowa Y, Poulikakos PI, Rosen N, Aoki Y, Sakai T (2013). Enhanced inhibition of ERK signaling by a novel allosteric MEK inhibitor, CH5126766, that suppresses feedback reactivation of RAF activity. Cancer Res.

[130] Valencia-Sama I, Ladumor Y, Kee L, Adderley T, Christopher G, Robinson CM, Kano Y, Ohh M, Irwin MS (2020). NRAS status determines sensitivity to SHP2 inhibitor combination therapies targeting the RAS-MAPK pathway in neuroblastoma. Cancer Res.

[131] King D, Yeomanson D, Bryant HE (2015). PI3King the lock: targeting the PI3K/Akt/mTOR pathway as a novel therapeutic strategy in neuroblastoma. J Pediatr Hematol Oncol.

[132] Mendoza MC, Er EE, Blenis J (2011). The Ras-ERK and PI3K-mTOR pathways: cross-talk and compensation. Trends Biochem Sci.

[133] Berry T, Luther W, Bhatnagar N, Jamin Y, Poon E, Sanda T, Pei D, Sharma B, Vetharoy WR, Hallsworth A (2012). The ALK(F1174L) mutation potentiates the oncogenic activity of MYCN in neuroblastoma. Cancer Cell.

[134] Moore NF, Azarova AM, Bhatnagar N, Ross KN, Drake LE, Frumm S, Liu QS, Christie AL, Sanda T, Chesler L (2014). Molecular rationale for the use of PI3K/AKT/mTOR pathway inhibitors in combination with crizotinib in ALK-mutated neuroblastoma. Oncotarget.

[135] Rader J, Russell MR, Hart LS, Nakazawa MS, Belcastro LT, Martinez D, Li Y, Carpenter EL, Attiyeh EF, Diskin SJ (2013). Dual CDK4/CDK6 inhibition induces cell-cycle arrest and senescence in neuroblastoma. Clin Cancer Res.

[136] Wood AC, Krytska K, Ryles HT, Infarinato NR, Sano R, Hansel TD, Hart LS, King FJ, Smith TR, Ainscow E (2017). Dual ALK and CDK4/6 inhibition demonstrates synergy against neuroblastoma. Clin Cancer Res.

[137] Wang HQ, Halilovic E, Li X, Liang J, Cao Y, Rakiec DP, et al. Combined ALK and MDM2 inhibition increases antitumor activity and overcomes resistance in human ALK mutant neuroblastoma cell lines and xenograft models. Elife. 2017;6. 10.7554/eLife.17137. 10.7554/eLife.17137 PMC5435462 28425916

[138] Coggins GE, Farrel A, Rathi KS, Hayes CM, Scolaro L, Rokita JL, Maris JM (2019). YAP1 mediates resistance to MEK1/2 inhibition in neuroblastomas with hyperactivated RAS signaling. Cancer Res.

[139] Subramonian D, Phanhthilath N, Rinehardt H, Flynn S, Huo Y, Zhang J, Messer K, Mo Q, Huang S, Lesperance J (2020). Regorafenib is effective against neuroblastoma in vitro and in vivo and inhibits the RAS/MAPK, PI3K/Akt/mTOR and Fos/Jun pathways. Br J Cancer.

