# Supplementary material for: A review of the biological and clinical implications of RAS-MAPK pathway alterations in neuroblastoma
Source: J Exp Clin Cancer Res. 2021 Jun 8;40:189. doi: 10.1186/s13046-021-01967-x (PMC8188681; doi:10.1186/s13046-021-01967-x)
Supplement: Supplementary file 1 — Additional file 1. [file 13046_2021_1967_MOESM1_ESM.docx]

**Gene References**

CASC14 / NBAT-1 [1, 2]

BARD1 [3-6]

LMO1 [7, 8]

DUSP12 [9-12]

LIN28B [13, 14]

HACE1 [13, 15, 16]

MLF1 / RSRC1 [17]

CDKN1B [16, 18-22]

SLC16A1 [23-27]

MSX1 [28, 29]

MMP20 [30]

KIF15 [31-33]

CHEK15 [5, 34, 35]

BRCA2 [36-39]

SMARCA4 [40-42]

LZTR1 [23, 25, 26, 43, 44]

BRCA1 [45-48]

APC [39, 49]

TP53 [50-83]

**References**

1. Pandey, G.K., et al., *The risk-associated long noncoding RNA NBAT-1 controls neuroblastoma progression by regulating cell proliferation and neuronal differentiation.* Cancer Cell, 2014. **26**(5): p. 722-37.

2. Yan, C., et al., *Long noncoding RNA NBAT-1 suppresses tumorigenesis and predicts favorable prognosis in ovarian cancer.* Onco Targets Ther, 2017. **10**: p. 1993-2002.

3. Capasso, M., et al., *Common variations in BARD1 influence susceptibility to high-risk neuroblastoma.* Nat Genet, 2009. **41**(6): p. 718-23.

4. Cote, D., et al., *Germline single nucleotide polymorphisms in ERBB3 and BARD1 genes result in a worse relapse free survival response for HER2-positive breast cancer patients treated with adjuvant based docetaxel, carboplatin and trastuzumab (TCH).* PLoS One, 2018. **13**(8): p. e0200996.

5. Lasorsa, V.A., et al., *Exome and deep sequencing of clinically aggressive neuroblastoma reveal somatic mutations that affect key pathways involved in cancer progression.* Oncotarget, 2016. **7**(16): p. 21840-52.

6. Woods, N.T., et al., *Charting the landscape of tandem BRCT domain-mediated protein interactions.* Sci Signal, 2012. **5**(242): p. rs6.

7. Wang, K., et al., *Integrative genomics identifies LMO1 as a neuroblastoma oncogene.* Nature, 2011. **469**(7329): p. 216-20.

8. Wang, L., et al., *ASCL1 is a MYCN- and LMO1-dependent member of the adrenergic neuroblastoma core regulatory circuitry.* Nat Commun, 2019. **10**(1): p. 5622.

9. Cho, S.S.L., et al., *Dual-Specificity Phosphatase 12 Targets p38 MAP Kinase to Regulate Macrophage Response to Intracellular Bacterial Infection.* Front Immunol, 2017. **8**: p. 1259.

10. Kumar, P., et al., *A Human Tyrosine Phosphatase Interactome Mapped by Proteomic Profiling.* J Proteome Res, 2017. **16**(8): p. 2789-2801.

11. Li, X., et al., *Defining the Protein-Protein Interaction Network of the Human Protein Tyrosine Phosphatase Family.* Mol Cell Proteomics, 2016. **15**(9): p. 3030-44.

12. Nguyen le, B., et al., *Phenotype restricted genome-wide association study using a gene-centric approach identifies three low-risk neuroblastoma susceptibility Loci.* PLoS Genet, 2011. **7**(3): p. e1002026.

13. Diskin, S.J., et al., *Common variation at 6q16 within HACE1 and LIN28B influences susceptibility to neuroblastoma.* Nat Genet, 2012. **44**(10): p. 1126-30.

14. Mizuno, R., et al., *Differential Regulation of LET-7 by LIN28B Isoform-Specific Functions.* Mol Cancer Res, 2018. **16**(3): p. 403-416.

15. Kumar, B., et al., *HACE1, an E3 Ubiquitin Protein Ligase, Mitigates Kaposi's Sarcoma-Associated Herpesvirus Infection-Induced Oxidative Stress by Promoting Nrf2 Activity.* J Virol, 2019. **93**(9).

16. Li, S., et al., *Mapping a dynamic innate immunity protein interaction network regulating type I interferon production.* Immunity, 2011. **35**(3): p. 426-40.

17. Taipale, M., et al., *A quantitative chaperone interaction network reveals the architecture of cellular protein homeostasis pathways.* Cell, 2014. **158**(2): p. 434-448.

18. Capasso, M., et al., *The functional variant rs34330 of CDKN1B is associated with risk of neuroblastoma.* J Cell Mol Med, 2017. **21**(12): p. 3224-3230.

19. Harkiolaki, M., et al., *Distinct binding modes of two epitopes in Gab2 that interact with the SH3C domain of Grb2.* Structure, 2009. **17**(6): p. 809-22.

20. Moeller, S.J., E.D. Head, and R.J. Sheaff, *p27Kip1 inhibition of GRB2-SOS formation can regulate Ras activation.* Mol Cell Biol, 2003. **23**(11): p. 3735-52.

21. Sugiyama, Y., et al., *Direct binding of the signal-transducing adaptor Grb2 facilitates down-regulation of the cyclin-dependent kinase inhibitor p27Kip1.* J Biol Chem, 2001. **276**(15): p. 12084-90.

22. Wolf, G., et al., *Erk 1,2 phosphorylates p27(Kip1): Functional evidence for a role in high glucose-induced hypertrophy of mesangial cells.* Diabetologia, 2003. **46**(8): p. 1090-9.

23. Adhikari, H. and C.M. Counter, *Interrogating the protein interactomes of RAS isoforms identifies PIP5K1A as a KRAS-specific vulnerability.* Nat Commun, 2018. **9**(1): p. 3646.

24. Avitabile, M., et al., *Neural crest-derived tumor neuroblastoma and melanoma share 1p13.2 as susceptibility locus that shows a long-range interaction with the SLC16A1 gene.* Carcinogenesis, 2020. **41**(3): p. 284-295.

25. Bigenzahn, J.W., et al., *LZTR1 is a regulator of RAS ubiquitination and signaling.* Science, 2018. **362**(6419): p. 1171-1177.

26. Kovalski, J.R., et al., *The Functional Proximal Proteome of Oncogenic Ras Includes mTORC2.* Mol Cell, 2019. **73**(4): p. 830-844 e12.

27. Liu, H.Y., et al., *lncRNA SLC16A1-AS1 as a novel prognostic biomarker in non-small cell lung cancer.* J Investig Med, 2020. **68**(1): p. 52-59.

28. Testori, A., et al., *Exploring Shared Susceptibility between Two Neural Crest Cells Originating Conditions: Neuroblastoma and Congenital Heart Disease.* Genes (Basel), 2019. **10**(9).

29. Xin, T., et al., *A novel mutation of MSX1 in oligodontia inhibits odontogenesis of dental pulp stem cells via the ERK pathway.* Stem Cell Res Ther, 2018. **9**(1): p. 221.

30. Chang, X., et al., *Common variants in MMP20 at 11q22.2 predispose to 11q deletion and neuroblastoma risk.* Nat Commun, 2017. **8**(1): p. 569.

31. Hungate, E.A., et al., *Evaluation of Genetic Predisposition for MYCN-Amplified Neuroblastoma.* J Natl Cancer Inst, 2017. **109**(10).

32. Wang, J., et al., *KIF15 promotes pancreatic cancer proliferation via the MEK-ERK signalling pathway.* Br J Cancer, 2017. **117**(2): p. 245-255.

33. Zhao, H., et al., *KIF15 promotes bladder cancer proliferation via the MEK-ERK signaling pathway.* Cancer Manag Res, 2019. **11**: p. 1857-1868.

34. Dai, B., et al., *Functional and molecular interactions between ERK and CHK2 in diffuse large B-cell lymphoma.* Nat Commun, 2011. **2**: p. 402.

35. Pugh, T.J., et al., *The genetic landscape of high-risk neuroblastoma.* Nat Genet, 2013. **45**(3): p. 279-84.

36. Moro, L., et al., *Constitutive activation of MAPK/ERK inhibits prostate cancer cell proliferation through upregulation of BRCA2.* Int J Oncol, 2007. **30**(1): p. 217-24.

37. Moro, L., et al., *Loss of BRCA2 promotes prostate cancer cell invasion through up-regulation of matrix metalloproteinase-9.* Cancer Sci, 2008. **99**(3): p. 553-63.

38. Wu, C., et al., *Systematic identification of SH3 domain-mediated human protein-protein interactions by peptide array target screening.* Proteomics, 2007. **7**(11): p. 1775-85.

39. Zhang, J., et al., *Germline Mutations in Predisposition Genes in Pediatric Cancer.* N Engl J Med, 2015. **373**(24): p. 2336-2346.

40. Esposito, M.R., et al., *Somatic mutations in specific and connected subpathways are associated with short neuroblastoma patients' survival and indicate proteins targetable at onset of disease.* Int J Cancer, 2018. **143**(10): p. 2525-2536.

41. Hendricks, K.B., F. Shanahan, and E. Lees, *Role for BRG1 in cell cycle control and tumor suppression.* Mol Cell Biol, 2004. **24**(1): p. 362-76.

42. Zhao, L.H., et al., *BAF complex is closely related to and interacts with NF1/CTF and RNA polymerase II in gene transcriptional activation.* Acta Biochim Biophys Sin (Shanghai), 2005. **37**(7): p. 440-6.

43. Abe, T., et al., *LZTR1 facilitates polyubiquitination and degradation of RAS-GTPases.* Cell Death Differ, 2020. **27**(3): p. 1023-1035.

44. Steklov, M., et al., *Mutations in LZTR1 drive human disease by dysregulating RAS ubiquitination.* Science, 2018. **362**(6419): p. 1177-1182.

45. Maekawa, T., et al., *ATF-2 controls transcription of Maspin and GADD45 alpha genes independently from p53 to suppress mammary tumors.* Oncogene, 2008. **27**(8): p. 1045-54.

46. Rosen, E.M., S. Fan, and C. Isaacs, *BRCA1 in hormonal carcinogenesis: basic and clinical research.* Endocr Relat Cancer, 2005. **12**(3): p. 533-48.

47. Yan, Y., et al., *Gamma-irradiation-induced DNA damage checkpoint activation involves feedback regulation between extracellular signal-regulated kinase 1/2 and BRCA1.* Cancer Res, 2008. **68**(13): p. 5113-21.

48. Yan, Y., et al., *BRCA1-induced apoptosis involves inactivation of ERK1/2 activities.* J Biol Chem, 2002. **277**(36): p. 33422-30.

49. Arroyo, R., et al., *Charting the molecular links between driver and susceptibility genes in colorectal cancer.* Biochem Biophys Res Commun, 2014. **445**(4): p. 734-8.

50. Aylon, Y., et al., *The Lats2 tumor suppressor augments p53-mediated apoptosis by promoting the nuclear proapoptotic function of ASPP1.* Genes Dev, 2010. **24**(21): p. 2420-9.

51. Bao, W. and S. Stromblad, *Integrin alphav-mediated inactivation of p53 controls a MEK1-dependent melanoma cell survival pathway in three-dimensional collagen.* J Cell Biol, 2004. **167**(4): p. 745-56.

52. Bulavin, D.V., et al., *Loss of oncogenic H-ras-induced cell cycle arrest and p38 mitogen-activated protein kinase activation by disruption of Gadd45a.* Mol Cell Biol, 2003. **23**(11): p. 3859-71.

53. Carlson, H., et al., *Tbx3 impinges on the p53 pathway to suppress apoptosis, facilitate cell transformation and block myogenic differentiation.* Oncogene, 2002. **21**(24): p. 3827-35.

54. Chen, T.C., et al., *Using an in situ proximity ligation assay to systematically profile endogenous protein-protein interactions in a pathway network.* J Proteome Res, 2014. **13**(12): p. 5339-46.

55. Chipps, E., et al., *Nuclear Localization Signal and p53 Binding Site in MAP/ERK Kinase Kinase 1 (MEKK1).* J Cell Biochem, 2015. **116**(12): p. 2903-14.

56. Cho, Y.Y., et al., *The p53 protein is a novel substrate of ribosomal S6 kinase 2 and a critical intermediary for ribosomal S6 kinase 2 and histone H3 interaction.* Cancer Res, 2005. **65**(9): p. 3596-603.

57. de Bruijn, M.T., et al., *Oncogenic KRAS sensitises colorectal tumour cells to chemotherapy by p53-dependent induction of Noxa.* Br J Cancer, 2010. **102**(8): p. 1254-64.

58. Deguin-Chambon, V., et al., *Direct transactivation of c-Ha-Ras gene by p53: evidence for its involvement in p53 transactivation activity and p53-mediated apoptosis.* Oncogene, 2000. **19**(51): p. 5831-41.

59. Deng, Q., et al., *The ability of E1A to rescue ras-induced premature senescence and confer transformation relies on inactivation of both p300/CBP and Rb family proteins.* Cancer Res, 2005. **65**(18): p. 8298-307.

60. Fogeron, M.L., et al., *LGALS3BP regulates centriole biogenesis and centrosome hypertrophy in cancer cells.* Nat Commun, 2013. **4**: p. 1531.

61. Gong, X., et al., *UV-induced interaction between p38 MAPK and p53 serves as a molecular switch in determining cell fate.* FEBS Lett, 2010. **584**(23): p. 4711-6.

62. How, P.C. and D. Shields, *Tethering function of the caspase cleavage fragment of Golgi protein p115 promotes apoptosis via a p53-dependent pathway.* J Biol Chem, 2011. **286**(10): p. 8565-76.

63. Jeong, M.H., et al., *p19ras interacts with and activates p73 by involving the MDM2 protein.* J Biol Chem, 2006. **281**(13): p. 8707-15.

64. Kato, K., et al., *Contribution of estrogen receptor alpha to oncogenic K-Ras-mediated NIH3T3 cell transformation and its implication for escape from senescence by modulating the p53 pathway.* J Biol Chem, 2002. **277**(13): p. 11217-24.

65. Kim, C.S., et al., *p53 impairs endothelium-dependent vasomotor function through transcriptional upregulation of p66shc.* Circ Res, 2008. **103**(12): p. 1441-50.

66. Kim, H.D., T.S. Kim, and J. Kim, *Aberrant ribosome biogenesis activates c-Myc and ASK1 pathways resulting in p53-dependent G1 arrest.* Oncogene, 2011. **30**(30): p. 3317-27.

67. Lambert, J.M., et al., *Mutant p53 reactivation by PRIMA-1MET induces multiple signaling pathways converging on apoptosis.* Oncogene, 2010. **29**(9): p. 1329-38.

68. Li, Z., et al., *The OncoPPi network of cancer-focused protein-protein interactions to inform biological insights and therapeutic strategies.* Nat Commun, 2017. **8**: p. 14356.

69. Melnikova, V.O., et al., *Mutant p53 is constitutively phosphorylated at Serine 15 in UV-induced mouse skin tumors: involvement of ERK1/2 MAP kinase.* Oncogene, 2003. **22**(38): p. 5958-66.

70. Peeper, D.S., et al., *Escape from premature senescence is not sufficient for oncogenic transformation by Ras.* Nat Cell Biol, 2001. **3**(2): p. 198-203.

71. Persons, D.L., E.M. Yazlovitskaya, and J.C. Pelling, *Effect of extracellular signal-regulated kinase on p53 accumulation in response to cisplatin.* J Biol Chem, 2000. **275**(46): p. 35778-85.

72. Rivlin, N., et al., *Rescue of embryonic stem cells from cellular transformation by proteomic stabilization of mutant p53 and conversion into WT conformation.* Proc Natl Acad Sci U S A, 2014. **111**(19): p. 7006-11.

73. Sablina, A.A., et al., *p53 activation in response to microtubule disruption is mediated by integrin-Erk signaling.* Oncogene, 2001. **20**(8): p. 899-909.

74. She, Q.B., et al., *Resveratrol-induced activation of p53 and apoptosis is mediated by extracellular-signal-regulated protein kinases and p38 kinase.* Cancer Res, 2001. **61**(4): p. 1604-10.

75. She, Q.B., N. Chen, and Z. Dong, *ERKs and p38 kinase phosphorylate p53 protein at serine 15 in response to UV radiation.* J Biol Chem, 2000. **275**(27): p. 20444-9.

76. Shih, A., et al., *Thyroid hormone promotes serine phosphorylation of p53 by mitogen-activated protein kinase.* Biochemistry, 2001. **40**(9): p. 2870-8.

77. Srivastav, R.K., et al., *Monitoring protein-protein interactions in mammalian cells by trans-SUMOylation.* Biochem J, 2011. **438**(3): p. 495-503.

78. Su, W., et al., *miR-30 disrupts senescence and promotes cancer by targeting both p16(INK4A) and DNA damage pathways.* Oncogene, 2018. **37**(42): p. 5618-5632.

79. Wu, G.S., *The functional interactions between the p53 and MAPK signaling pathways.* Cancer Biol Ther, 2004. **3**(2): p. 156-61.

80. Yeh, P.Y., et al., *Phosphorylation of p53 on Thr55 by ERK2 is necessary for doxorubicin-induced p53 activation and cell death.* Oncogene, 2004. **23**(20): p. 3580-8.

81. Yeh, P.Y., et al., *Nuclear extracellular signal-regulated kinase 2 phosphorylates p53 at Thr55 in response to doxorubicin.* Biochem Biophys Res Commun, 2001. **284**(4): p. 880-6.

82. You, X., et al., *PUMA-mediated apoptosis in fibroblast-like synoviocytes does not require p53.* Arthritis Res Ther, 2006. **8**(6): p. R157.

83. Zhang, H., et al., *Nocodazole-induced p53-dependent c-Jun N-terminal kinase activation reduces apoptosis in human colon carcinoma HCT116 cells.* J Biol Chem, 2002. **277**(46): p. 43648-58.
